# Interactions between all pairs of neighboring trees in 16 forests worldwide reveal details of unique ecological processes in each forest, and provide windows into their evolutionary histories

**DOI:** 10.1371/journal.pcbi.1008853

**Published:** 2021-04-29

**Authors:** Christopher Wills, Bin Wang, Shuai Fang, Yunquan Wang, Yi Jin, James Lutz, Jill Thompson, Kyle E. Harms, Sandeep Pulla, Bonifacio Pasion, Sara Germain, Heming Liu, Joseph Smokey, Sheng-Hsin Su, Nathalie Butt, Chengjin Chu, George Chuyong, Chia-Hao Chang-Yang, H. S. Dattaraja, Stuart Davies, Sisira Ediriweera, Shameema Esufali, Christine Dawn Fletcher, Nimal Gunatilleke, Savi Gunatilleke, Chang-Fu Hsieh, Fangliang He, Stephen Hubbell, Zhanqing Hao, Akira Itoh, David Kenfack, Buhang Li, Xiankun Li, Keping Ma, Michael Morecroft, Xiangcheng Mi, Yadvinder Malhi, Perry Ong, Lillian Jennifer Rodriguez, H. S. Suresh, I Fang Sun, Raman Sukumar, Sylvester Tan, Duncan Thomas, Maria Uriarte, Xihua Wang, Xugao Wang, T. L. Yao, Jess Zimmermann

**Affiliations:** 1 Division of Biological Sciences, University of California, San Diego, La Jolla, California, United States of America; 2 Guangxi Key Laboratory of Plant Conservation and Restoration Ecology in Karst Terrain, Guangxi Institute of Botany, Guangxi Zhuang Autonomous Region and Chinese Academy of Sciences, Guilin; 3 Key Laboratory of Forest Ecology and Management, Institute of Applied Ecology, Chinese Academy of Sciences, Shenyang; 4 College of Chemistry and Life Sciences, Zhejiang Normal University, Jinhua; 5 State Key Laboratory of Vegetation and Environmental Change, Institute of Botany, Chinese Academy of Sciences, 20 Nanxincun, Xiangshan, Beijing; 6 College of Life Sciences, Zhejiang University, Hangzhou; 7 Department of Wildland Resources, Utah State University, Logan, Utah, United States of America; 8 Center for Ecology & Hydrology, Penicuik, Midlothian, Scotland; 9 Department of Biological Sciences, Louisiana State University, Baton Rouge, Los Angeles, United States of America; 10 Divecha Centre for Climate Change, Indian Institute of Science, Bengaluru, India; 11 National Centre for Biological Sciences, GKVK Campus, Bengaluru, India; 12 Center for Integrative Conservation, Xishuangbanna Tropical Botanical Garden, Chinese Academy of Sciences, Menglun, Mengla, Yunnan; 13 Zhejiang Tiantong Forest Ecosystem National Observation and Research Station, School of Ecological and Environmental Sciences, East China Normal University, Shanghai; 14 Department of Biology, Memorial University of Newfoundland, Newfoundland, Canada; 15 Institute of Ecology and Evolutionary Biology, National Taiwan University, Taipei; 16 School of Geography and the Environment, University of Oxford, Oxford, United Kingdom; 17 School of Biological Sciences, The University of Queensland, St. Lucia, Queensland, Australia; 18 Department of Ecology, State Key Laboratory of Biocontrol and School of Life Sciences, Sun Yat-sen University, Guangzhou; 19 Department of Botany and Plant Physiology, University of Buea, Cameroon; 20 Department of Biological Sciences, National Sun Yat-sen University, Kaohsiung; 21 National Centre for Biological Sciences, Bengaluru, India; 22 Center for Tropical Forest Science, Smithsonian Institution, Washington, DC, United States of America; 23 Faculty of Science and Technology, Uva Wellassa University, Badulla, Sri Lanka; 24 Department of Botany, University of Peradeniya, Peradeniya Sri Lanka; 25 Forest Research Institute Malaysia, Kepong Selangor, Malaysia; 26 Dept. of Botany, Faculty of Science, University of Peradeniya, Peradeniya Sri Lanka; 27 Taiwan Forestry Research Institute, Taipei; 28 Department of Ecology and Evolutionary Biology, University of California, Los Angeles, Los Angeles, California, United States of America; 29 Graduate School of Science, Osaka City University, Sumiyoshi Ku, Osaka, Japan; 30 Center for Tropical Forest Science–Forest Global Earth Observatory (CTFS-ForestGEO), Smithsonian Tropical Research Institute, NMNH—MRC, Washington, DC, United States of America; 31 Natural England Mail Hub, County Hall, Worcester, United Kingdom; 32 School of Geography and the Environment, Oxford University Centre for the Environment, University of Oxford, Oxford, United Kingdom; 33 Institute of Biology, College of Science, University of the Philippines Diliman, Diliman, Quezon City, Philippines; 34 Centre for Ecological Sciences, Indian Institute of Science, Bengaluru, India; 35 Department of Natural Resources and Environmental Studies, National Dong Hwa University, Hualien; 36 Forest Department Sarawak, Bangunan Wisma Sumber Alam, Jalan Stadium, Petra Jaya, Kuching, Sarawak, Malaysia; 37 Department of Biology, Washington State University, Vancouver, Washington State, United States of America; 38 Department of Ecology, Evolution, and Environmental Biology, Columbia University, New York city, New York, United States of America; 39 Dept of Environmental Sciences, University of Puerto Rico, Rio Piedras, San Juan, PR, United States of America; University of Chicago, UNITED STATES

## Abstract

When Darwin visited the Galapagos archipelago, he observed that, in spite of the islands’ physical similarity, members of species that had dispersed to them recently were beginning to diverge from each other. He postulated that these divergences must have resulted primarily from interactions with sets of other species that had also diverged across these otherwise similar islands. By extrapolation, if Darwin is correct, such complex interactions must be driving species divergences across all ecosystems. However, many current general ecological theories that predict observed distributions of species in ecosystems do not take the details of between-species interactions into account. Here we quantify, in sixteen forest diversity plots (FDPs) worldwide, highly significant negative density-dependent (NDD) components of both conspecific and heterospecific between-tree interactions that affect the trees’ distributions, growth, recruitment, and mortality. These interactions decline smoothly in significance with increasing physical distance between trees. They also tend to decline in significance with increasing phylogenetic distance between the trees, but each FDP exhibits its own unique pattern of exceptions to this overall decline. Unique patterns of between-species interactions in ecosystems, of the general type that Darwin postulated, are likely to have contributed to the exceptions. We test the power of our null-model method by using a deliberately modified data set, and show that the method easily identifies the modifications. We examine how some of the exceptions, at the Wind River (USA) FDP, reveal new details of a known allelopathic effect of one of the Wind River gymnosperm species. Finally, we explore how similar analyses can be used to investigate details of many types of interactions in these complex ecosystems, and can provide clues to the evolution of these interactions.

## Author summary

Worldwide, ecosystems are collapsing or in danger of collapse, but the precise causes of these collapses are often unknown. Observational and experimental evidence shows that all ecosystems are characterized by strong interactions between and among species, and that these webs of interactions can be important contributors to the preservation of ecosystem diversity. But many of the interactions–such as those involving pathogenic microorganisms and the chemical defenses that are mounted by their prey–are not easily identified and analyzed in ecosystems that may have hundreds or thousands of species. Here we use our equal-area-annulus analytical method to examine census data from over three million trees in forest plots from around the world. We show how the method can be used to flag pairs and groups of species that exhibit unusual levels of interaction and that are likely on further investigation to yield information about their causative mechanisms. We give a detailed example showing how some of these interactions can be traced to defense mechanisms that are possessed by one of the tree species. We explore how our method can be used to identify the between-species interactions that play the largest roles in the maintenance of ecosystems and their diversity.

## Introduction

The] inhabitants of each separate [Galapagos] island, though mostly distinct, are related in an incomparably closer degree to each other than to the inhabitants of any other part of the world‥‥ [Dissimilarities] between the endemic inhabitants of the islands may be used as an argument against my views; for it may be asked, how has it happened in the several islands situated within sight of each other, having the same geological nature, the same height, climate, &c., that many of the immigrants should have been differently modified, though only in a small degree. This long appeared to me a great difficulty: but it arises in chief part from the deeply-seated error of considering the physical conditions of a country as the most important for its inhabitants; whereas it cannot, I think, be disputed that the nature of the other inhabitants, with which each has to compete, is at least as important, and generally a far more important element of success.

Charles Darwin, *The Origin of Species*, 1st ed. 1859, p. 400.

In this passage from the *Origin*, Darwin effectively founded the field of evolutionary ecology. He was faced with the difficulty of explaining recent adaptive radiations that sometimes resulted in distinct species on the different islands of the Galápagos archipelago, even though the islands have similar physical properties. The solution, he suggested, must lie in these evolving populations’ interactions with other species, the mix of which should differ among the individual islands. (And those other species, by his reasoning, would simultaneously be evolving in their own unique directions as a result of their own sets of between-species interactions.) But his claim that the importance of such between-species interactions "cannot be disputed" was far from being demonstrated at the time.

In the century and a half since the *Origin*, ecologists and evolutionary biologists have explored the many interactions among species that share the same ecological community, in ever-greater detail and with ever-more-sophisticated tools [1]. Modeling has pointed the way [2–6]. Even so, such interactions must involve many more species, occupying a variety of different trophic levels, than those that can be examined in a typical study. Host-pathogen interactions were early postulated to be important in the maintenance of species diversity [7], and were soon realized to have a high likelihood of contributing to negative density-dependent (NDD) interactions between host species [8, 9]. Such interactions have been detected in the relatively simple ecosystems of the Galápagos [10] and in complex ecosystems such as tropical forests [11–13], coral reefs [14], and lacustrine fish communities [15].

The classic Lotka-Volterra model, based on competition coefficients, examines species that compete directly for resources, and shows that the species can coexist if each has resources that other species cannot access regardless of their numbers [2]. A second important group of models involves NDD interactions, in which a selective advantage to species that are locally rare switches to a selective disadvantage when those species become locally common. NDD can lead to multiple stable internal density equilibria that permit numerous species to occupy the same ecosystem [3–6]. Possible mechanisms for NDD effects can include species interactions with both physical and biological factors.

Many general ecological theories seeking to explain the distributions of species that occupy the same or similar trophic levels have tended to gloss over such complexities. In 2010, McGill [16] surveyed six “unified theories of biodiversity,” all of which had shown success in predicting observed species abundance distributions and species-area curves at scales of 100 m and above. He showed that all these theories employ three assumptions: intraspecific clumping, intraspecific variation in global abundance, and—most importantly for the present study—spatial independence of the distributions of different coexisting species.

Darwin had postulated that species populating a multitude of trophic levels in an ecosystem are continuously interacting, and that these interactions contribute to evolutionary divergence. Given the growing evidence for such interactions (see [17] for an extreme example), it is surprising that apparently successful general ecological theories can be constructed using the assumption that species-species interactions are irrelevant to the overall structure of communities. General unified theories of ecosystems may indeed be congruent with the distributions of component species that happen to be easily countable, provided that the scale is 100 m and above. But they incorporate no information about the existence of biotic and abiotic interactions at smaller scales, which is where most between-species interactions are likely to take place.

How common, how complex, and how significant in their effects are the fine-structure between-species interactions that Darwin postulated? Can an understanding of these interactions lead to more complete theories that underlie ecosystem structures and their evolutionary trajectories? Here we test the spatial independence assumption that McGill shows is basic to the most general ecological distribution theories. We examine sixteen multiply-censused forest diversity plots (FDPs) that are scattered over a wide variety of biogeographic regions (Table 1), and find that the assumption does not hold at the scale of meters. We also show that the pattern of departures from independence can reveal new information about between-species interactions.

**Table 1:** Some characteristics of the FDPs examined in this paper, arranged by latitude.

FDP	Dim (m)	No. of census intervals	Total of species recorded	Avg. tree density (trees/m2) & no. annuli used	Annual Rainfall (mm)	Latitude/Longitude	Min/max or avg. temp (oC)
Pasoh (Peninsular Malaysia)	1000 X 500	5	898	0.671 20	2000	2.98N/102.3E	25.8/28.3
Lambir (Sarawak, Malaysia)	1040x500	2	1180	0.665 10	2700	4.2N/114E	31.4/22.1
Korup (Cameroon)	1000 x 500	2	494	0.656 10	5500 (seasonal)	5.1N/8.9E	22.7/30.6
Sinharaja (Sri Lanka)	500 x 500	2	239	0.829 10	5000	6.4N/80.4E	20.4/24.7
Barro Colorado Island (Panama)	1000 x 500	6	320	0.470 20	2600 (seasonal)	9.15N/79.85W	23/32
Mudumalai (India)	1000 x 500	2 (4-yr intervals)	76	0.035 10	1300 (seasonal)	11.6N/76.5E	16.4/27.4
Palanan (Philippines)	400 x 400	3	319	0.210 10	3200 (typhoons)	17.0N/122.4E	26.1
Luquillo (Puerto Rico)	320x500	4	163	0.289 10	3500 (hurricanes)	18.3N/65.8W	23.0
Nonggang	500 x 300	1	217	0.453 10	1300 (seasonal)	22.4N/107.0E	19.0/27.2
Heishiding	1000 x 500	1	245	0.546 10	1700 (seasonal)	23.3N/111.5E	10.6/28.4
Fushan	500 x 500	1	111	0.463 10	4300 (typhoons)	24.8N/121.6E	18.2
Gutianshan	600 x 400	2	159	0.586 10	2000	29.1N/118.1E	4.3/27.9
Tiantong	500 x 400	1	154	0.604 10	5000 (some typhoons)	29.8N/121.8E	16.2
Changbaishan	500 x 500	2	52	0.155 10	700	42.4N/128.1E	3.6
Wind River (US)	800 x 340	1	26	0.116 10	2300 (seasonal)	45.8N/122W	-2/27
Wytham Woods (UK)	300x600	2	24	0.112 10	700	51.8N/1.34W	10

We use the Equal-Area Annulus (EAA) [18] point-pattern method to visualize and quantify non-random patterns of tree clustering, distributions of tree recruitment and mortality, and the influence of surrounding trees on tree growth. We show details of how these interactions occur not only between conspecifics, where they are well-known [5, 13, 19, 20], but also between species that are separated across a wide range of phylogenetic distances. We show that, although the interactions decrease smoothly in significance with increasing physical distance between trees, they exhibit complex relationships with phylogenetic distance that are unique to each of the study’s FDPs. Such complexities are not dealt with in the global theories examined by McGill.

Because of the many differences between EAA and the commonly-used regression-based methods that are used to detect NDD effects, and because of the many modifications that have been made to EAA since its original publication (subheads 1–8 in the Modifications to the Original Method section), we have chosen to place the extensive Materials and Methods section immediately following this introduction. We emphasize how the EAA method avoids the statistical biases [21] that are inherent in regression-based methods. The "regression dilution" issue flagged by that paper is not a problem in our analyses because a bias towards zero makes the EAA method less—not more—likely to detect NDD (i.e. errors in predictors would reduce the statistical power of the method instead of increasing the Type I error rate).

The EAA method also incorporates a number of desirable features that ideally should be exhibited by methods designed to detect density-dependent effects, as discussed in a recent review [22]. A method should (1) measure the relative magnitudes of conspecific NDD and heterospecific NDD and how they vary among different between-species interactions, (2) evaluate the relative roles of conspecific and heterospecific NDD in the maintenance of ecological diversity, (3) remove the biases inherent in statistical methods that do not compare the actual data with appropriate null models, (4) distinguish the relative sizes of the contributions to NDD of species with different abundances and life histories, and the contributions of biogeographic factors such as latitude and rainfall, (5) follow the contributions of organisms at different stages in their life histories, (6) provide a route for further examination of the details of the NDD mechanisms themselves and the long-term evolutionary implications of these mechanisms.

**Fig 1:**
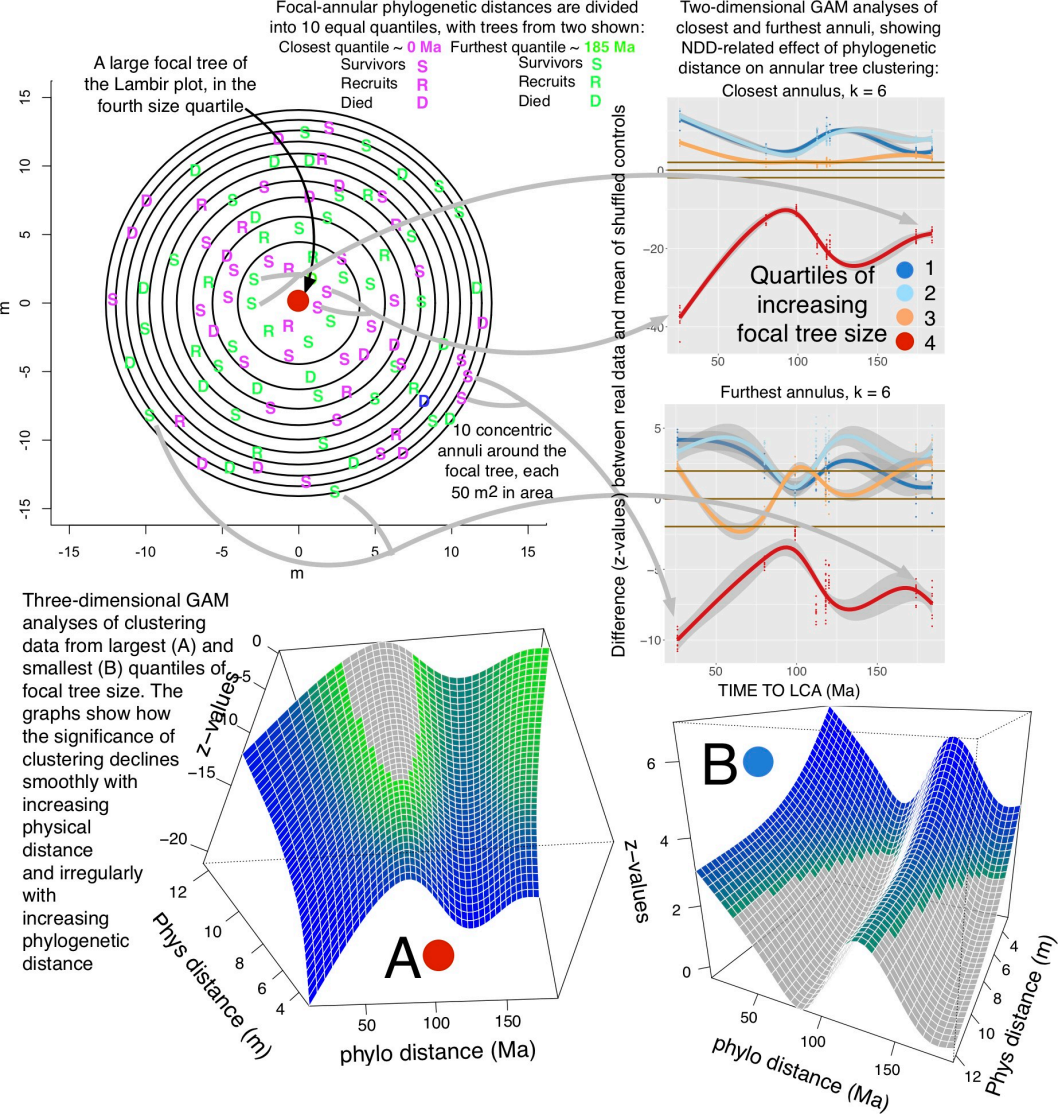
An overview of a typical EAA analysis. At top is a diagram of a large focal tree in the Lambir (Malaysia) FDP, surrounded by 10 concentric annuli each of area 50 m^2^. For simplicity, the trees shown in the diagram are only a sample of some of the annular surviving trees (S), recruits (R), and trees that die (D). In the diagram the trees shown are sampled from among the trees that lie at zero or at 185 Ma (mega-annum) phylogenetic distances from their LCA with the focal tree, although of course all the trees in the annuli are used in the entire EAA analysis. Generalized Additive Model (GAM) fits to patterns of clustering of surviving annular trees, using data from the closest and the furthest annulus, are shown in the two-dimensional graphs on the right of the diagram. In this analysis, the observed annular clustering is presented as deviations (z-values) from a null model expectation for four quantiles of focal tree diameter. The null model is generated by repeated shuffling of the focal tree diameters within species, so that any positive z-values for some of the focal-annular size quantiles must be balanced by negative values for others. Such positive-negative balances are expected in analyses of recruitment, clustering and mortality, but not in analyses of growth (Materials and Methods). The 95% confidence intervals of the GAM curves are shown in gray. Brown horizontal lines show the 95% confidence intervals around zero z-values. To help orient the viewer, gray arrows connect some of the closely-related and distantly-related survivors in the diagram to the places at which their data contributes to the largest-quantile focal tree lines (red) on the two-dimensional graphs. Each data point in the 2D graphs represents the difference between the actual and the null-model data for all focal trees in a given census period that have annular trees within a specific phylogenetic range. The null-model data have been generated by repeated shuffling of focal tree properties (size or growth rate) within species. A new point is generated for each of the ten replicates of the actual-null comparisons and for each of the census periods at the FDP. The points in the graphs form clusters because, with each replicate, species pairs separated by similar phylogenetic distances are shuffled at random in order to fill each of the phylogenetic distance quantiles. Thus, each of the gray arrows that shows the contribution of an individual tree simply shows where the tiny amount of information that is contributed by that tree’s focal-annular interactions ends up in the data points in the graphs. The three-dimensional graphs show GAM fits of the largest-quantile (graph A) and smallest-quantile (graph B) focal tree size data across all ten annuli. Regions of the surfaces that lie within the range of non-significant z-values along the z-axis are gray; those that lie outside this range, and that therefore represent significant z-values, are colored. The colors start with green and shade through blue as the significance of the positive or negative z-values increases. The orientations of the three-dimensional graphs presented here sometimes differ, in order to reveal details of the surfaces. As with the lines on the two-dimensional graphs, the 3D surfaces themselves have confidence intervals, but the confidence intervals are not shown here. Typical confidence intervals on the 3D surfaces, which tend to be small, are visualized more easily if these three-dimensional graphs can be rotated by the viewer. A sampling of such rotatable graphs is presented as html files in S3–S11 Figs.

**Fig 2:**
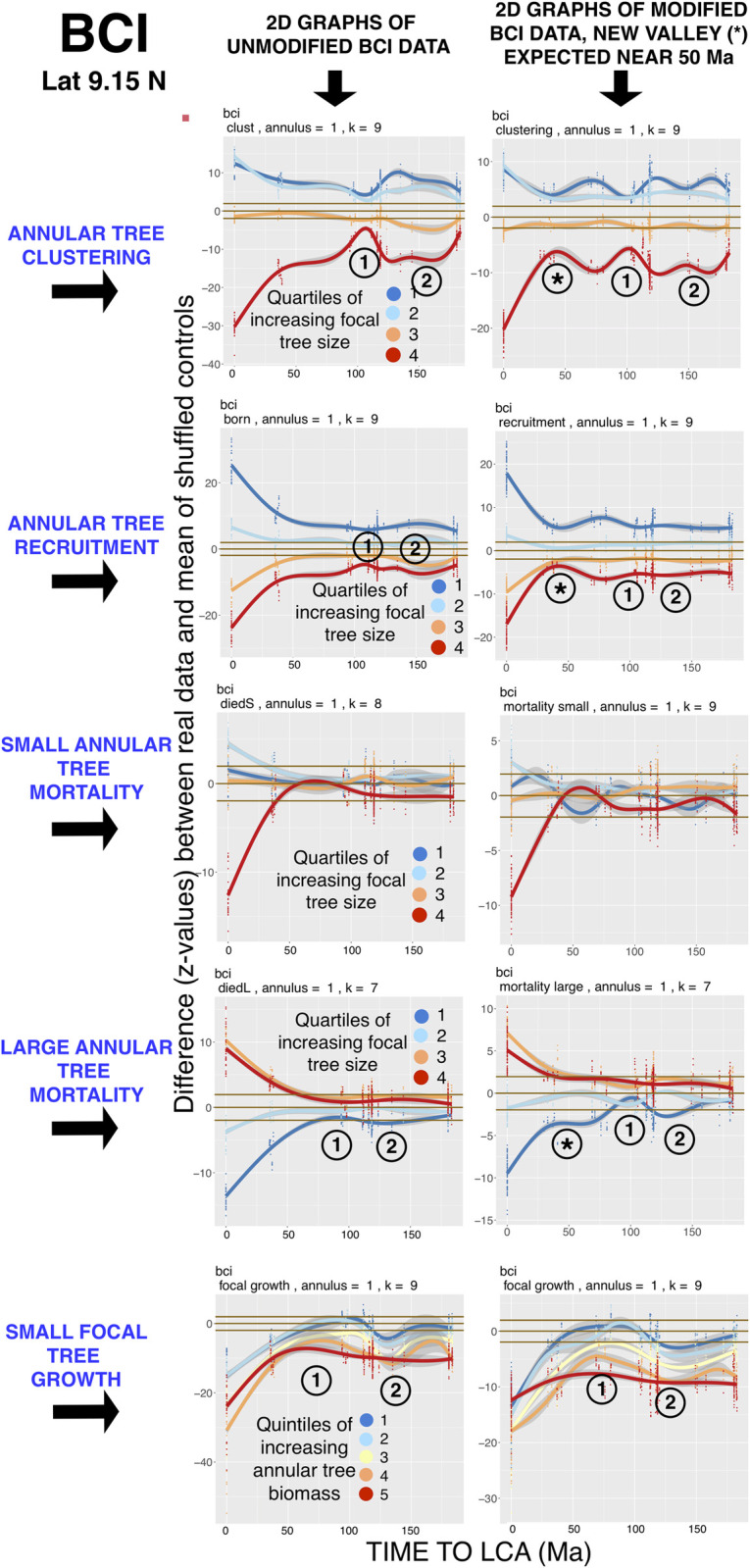
Test of the sensitivity of EAA analysis. The circled 1 and 2 mark unusual valleys and peaks respectively in the significance of focal-annular interactions. Asterisks mark the appearance of a new “valley” in significance levels after deliberate manipulation of the data (see text). Other legends as in Fig 1.

Unlike regression-based methods, EAA uses null models that isolate the variables being examined while leaving all the other properties of these extensive data sets unchanged. This enables us to present details of the species-species interactions with high statistical confidence. For clarity, we provide a step-by-step illustration of an EAA analysis (Fig 1). We also provide an example showing that the method is highly sensitive to small deliberately-introduced changes in the FDP data (subhead 11 of the Modifications to the Original Method section and Fig 2).

**Fig 3:**
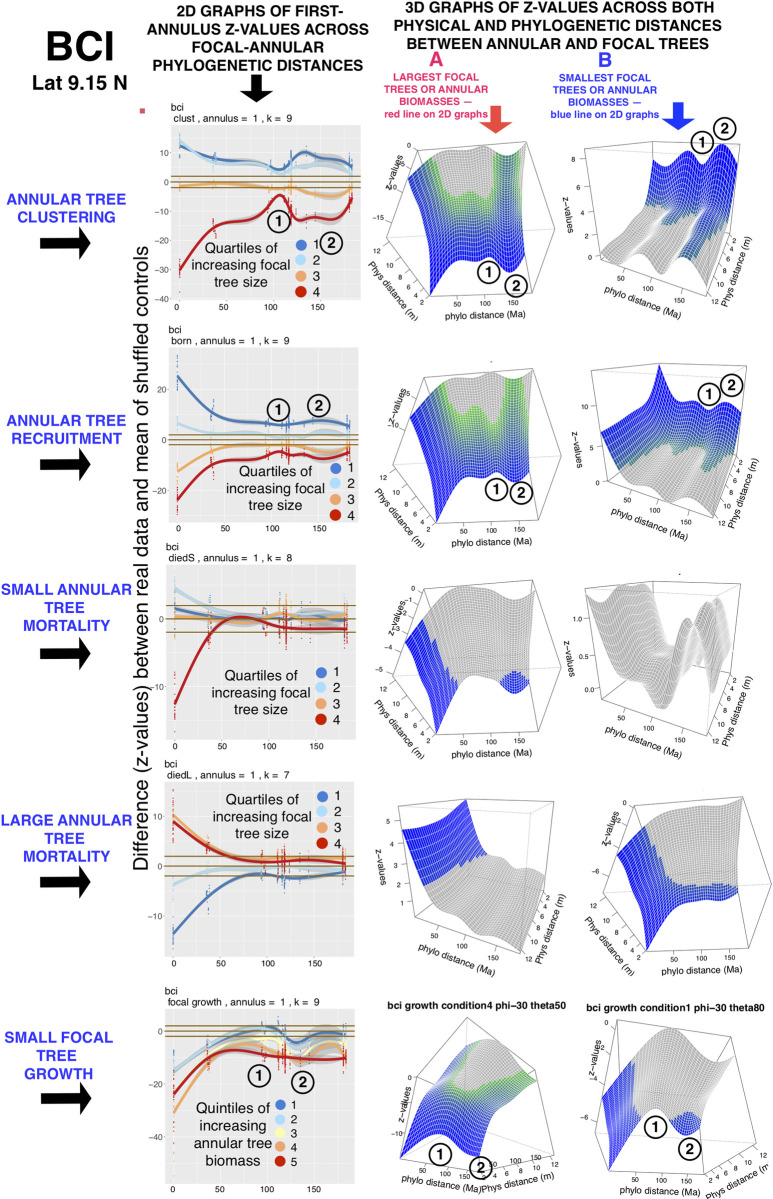
A comparison of two- and three-dimensional graphs of the z-values for NDD-influenced focal-annular patterns at the BCI (Panama) FDP. The 2D graphs show first-annulus z-values of these patterns for all subdivisions of focal tree sizes and annular tree biomasses, compared to null models (Materials and Methods). The 3D graphs show the surfaces formed by the z-values across both physical and phylogenetic distances between focal and annular trees. The surfaces shown are for the largest focal trees or annular biomasses (red lines in the 2D graphs) and the smallest focal trees or annular biomasses (blue lines in the 2D graphs). Circled numerals 1 and 2 mark unusually low or high z-values that are found at certain focal-annular phylogenetic distances. The k-values are the optimized number of smooth terms used in the GAM analyses (Materials and Methods). The 95% confidence limits for the expected 2D z-values of zero are shown as brown horizontal lines, and the 95% confidence intervals of the 2D lines are shown in gray. The regions of significant z-values on the 3D surfaces are heat-map colored as in Fig 1, and the non-significant regions are gray.

**Fig 4:**
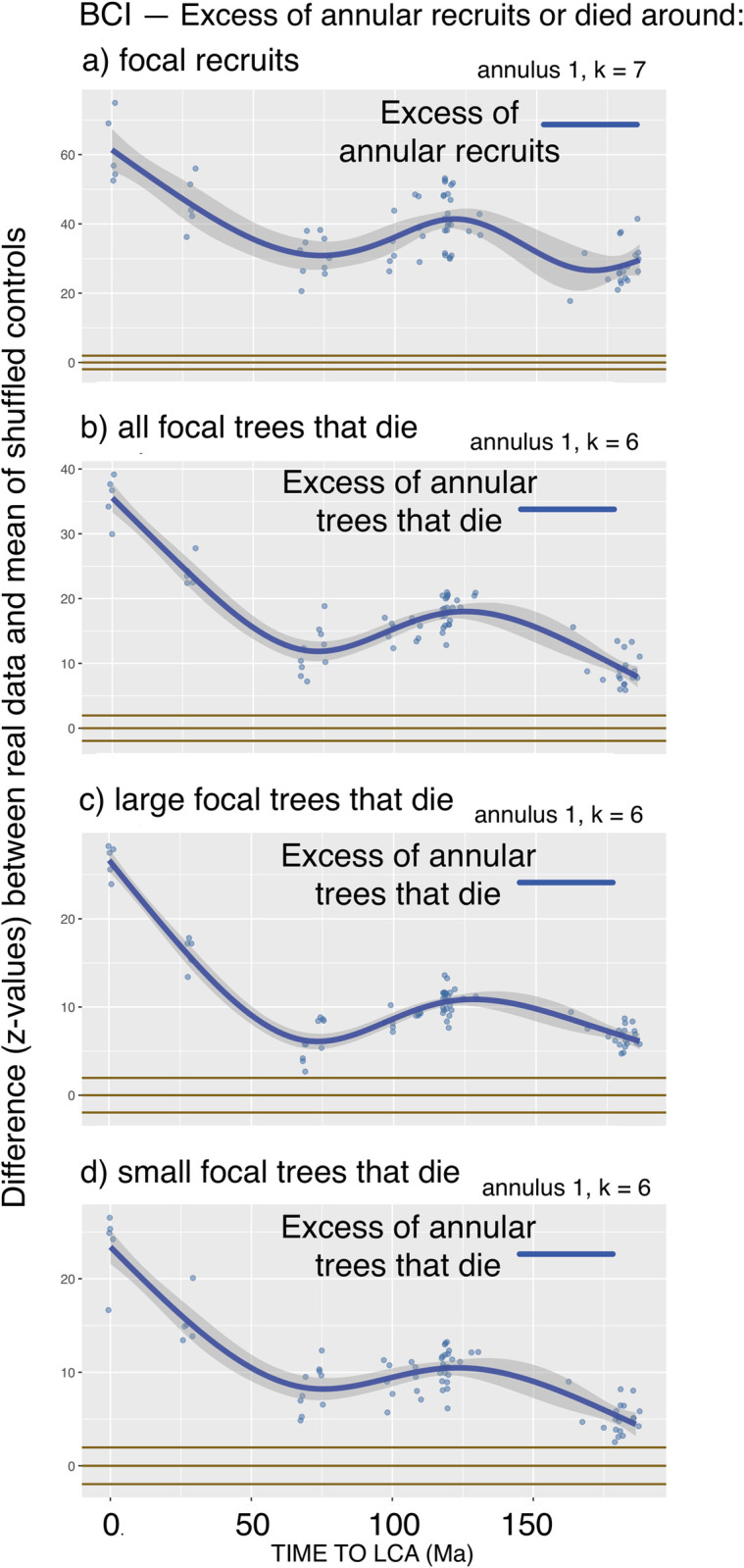
Tests at the BCI FDP of the proportions of recruits or trees that die in Annulus 1 around focal recruits or focal trees that die, compared with the proportions expected from a null model in which the properties (recruits, survivors, died) of the focal trees are shuffled repeatedly within species.

**Fig 5:**
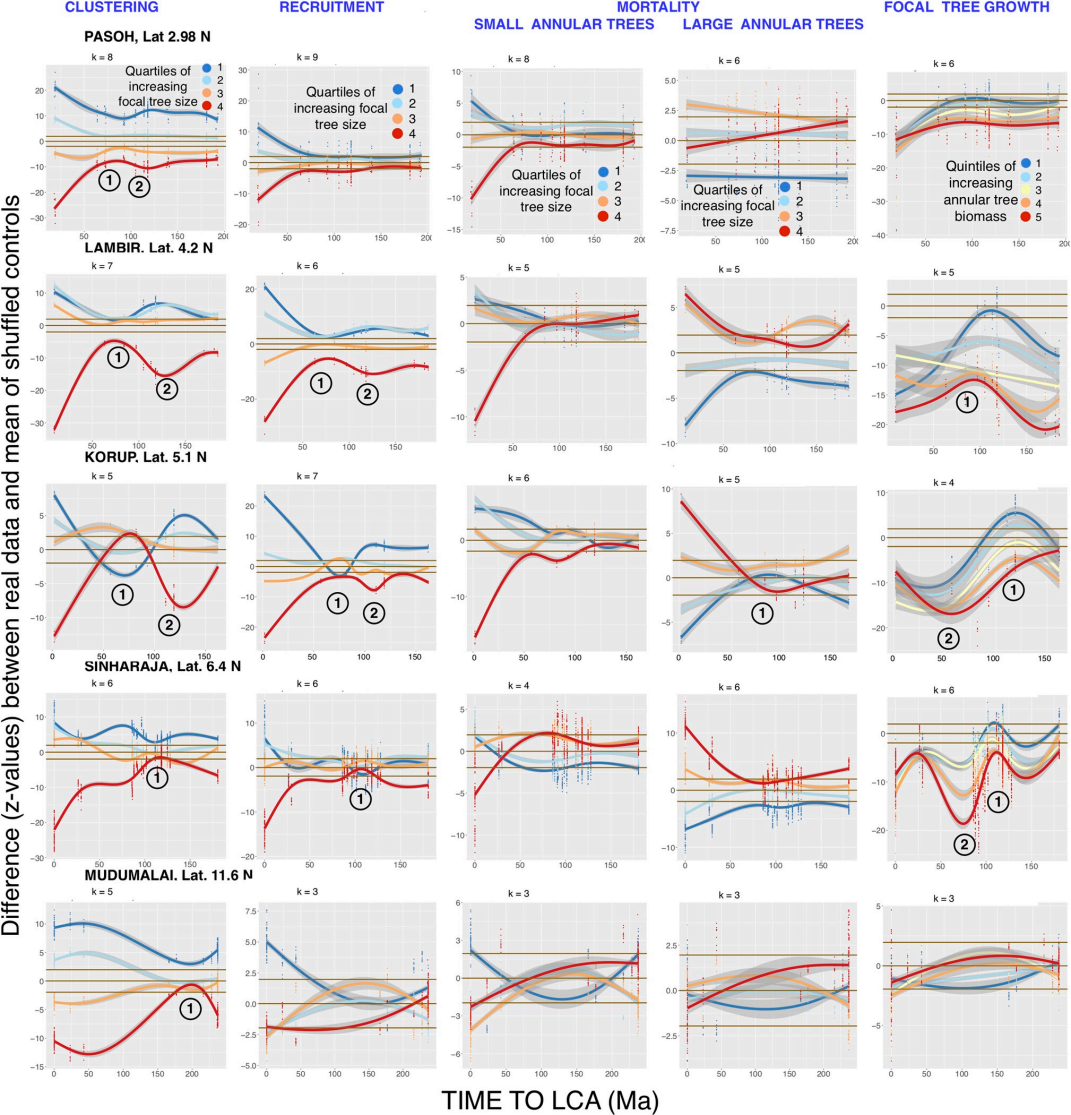
This figure and the following two figures show two-dimensional GAM analyses of NDD-influenced focal-annular interactions for the remaining fifteen FDPs in the study (excluding BCI, which is presented in Fig 3). This figure shows results from the lowest-latitude FDPs. Legends as in Figs 1 and 3. The k-values are the optimized number of smooth terms. Circled numbers 1 and 2 represent local reductions or increases respectively in the significance of the effects along the phylogenetic distance axis.

**Fig 6:**
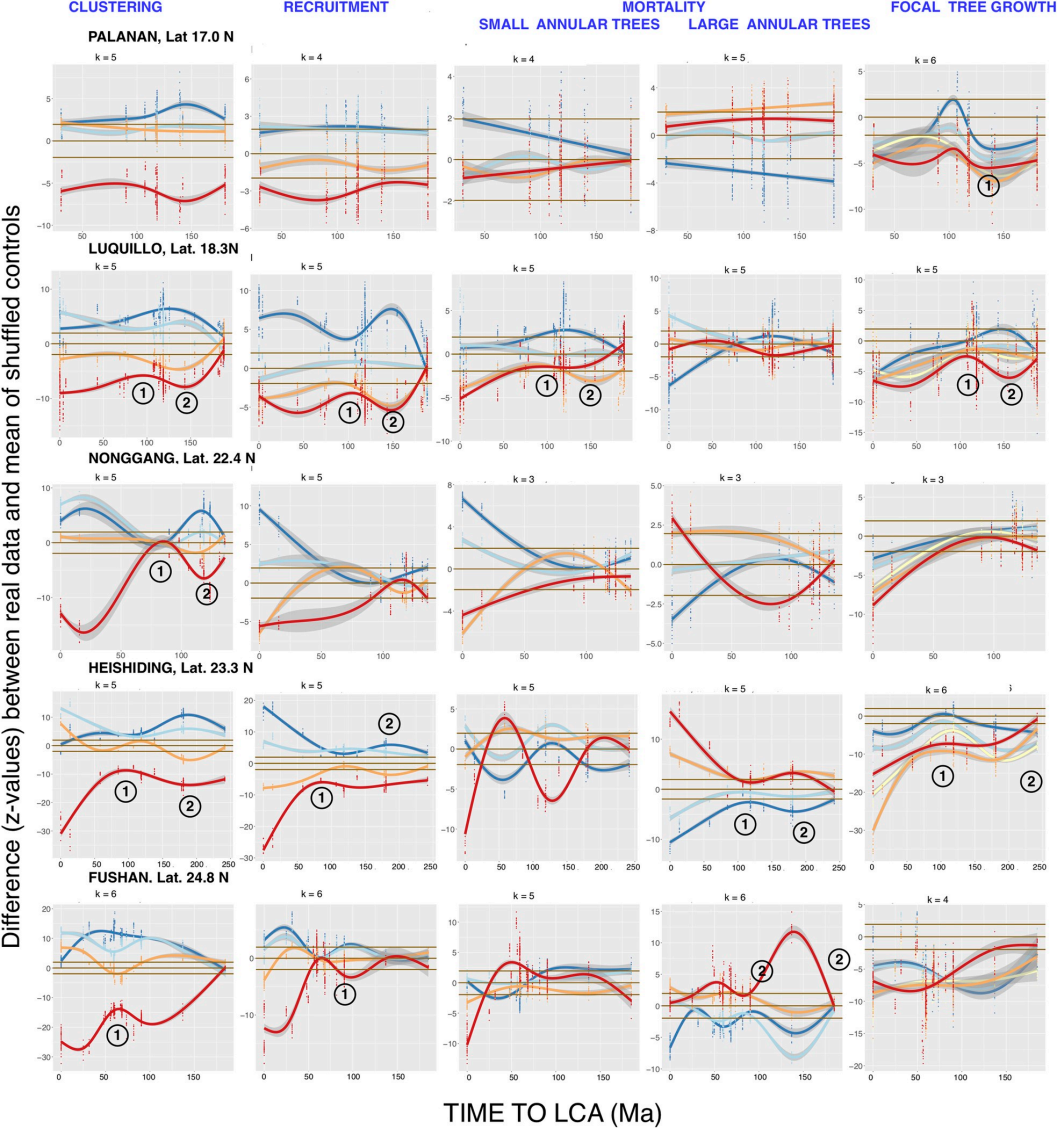
Continuation of the first-annulus two-dimensional phylogenetic distance analyses of all the FDPs, showing FDPs at intermediate latitudes. Legends as in Figs 1 and 3.

**Fig 7:**
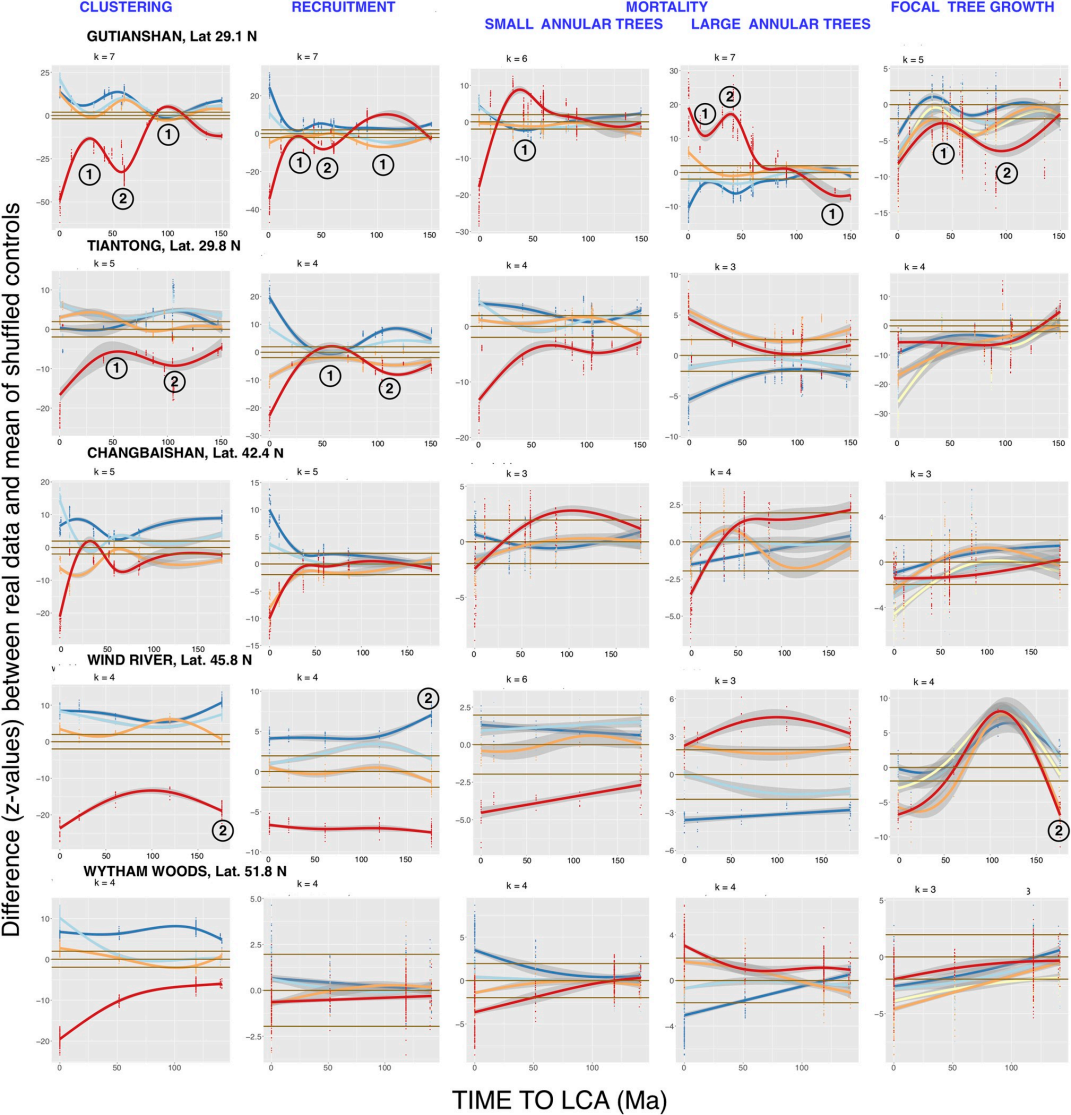
The highest-latitude set of the first-annulus two-dimensional phylogenetic distance analyses of all the FDPs. Legends as in Figs 1 and 3.

**Fig 8:**
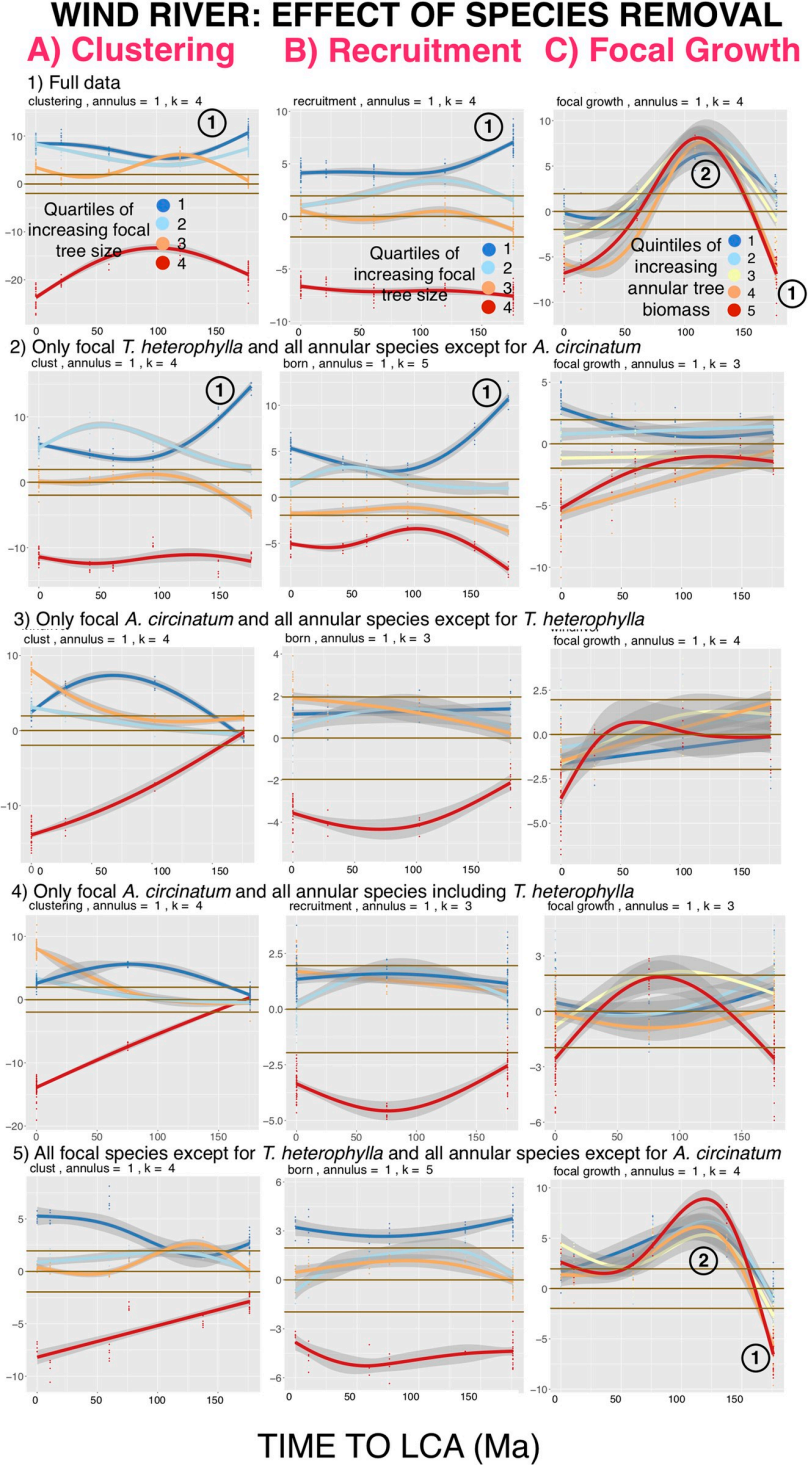
EAA two-dimensional first-annulus analysis of allelopathic effects of *Tsuga heterophylla* on other species in the Wind River FDP. Three-dimensional analyses (not shown) show smooth declines in significance with increasing physical distance but preservation of localized phylogenetic distance features across annuli. See text for interpretation. Legends and confidence intervals as in Fig 1.

In the Results section, we present EAA analyses of all sixteen FDPs (Figs 3–7), and examine in detail the causes of the heterospecific interaction peaks and valleys that are observed at the temperate Wind River (Washington State, USA) FDP (Fig 8). We show how EAA analysis provides new details of the role played by strong allelopathic effects of a gymnosperm, the western hemlock *Tsuga heterophylla*, on some but not all of the angiosperms in the FDP to which it is very distantly related [23]. This example demonstrates the potential of EAA to examine the roles of heterospecific interactions that may involve a wide variety of tree characteristics and microenvironmental factors. EAA provides a tool to measure the sizes of the contributions of these variables to species distributions and life history patterns. Our preliminary findings show that EAA results and the experiments that they suggest will help to pinpoint unusually significant interactions that can be investigated further through field observations and experiments. These findings will in turn enable us to unravel the true complexity and the evolutionary histories of the many between-species interactions that, as Darwin had believed, “cannot be disputed.”

## Materials and methods

### FDP data

This paper surveys demographic and tree-distribution data from 16 repeatedly-censused forest dynamics plots (FDPs) (https://forestgeo.si.edu/sites-all). The FDPs have been established in locations ranging from tropical to high-temperate latitudes, and encompass a wide range of seasonal and non-seasonal rainfall patterns (Table 1). Repeated censuses of the FDPs include all trees present during each census that have a diameter of 1 cm or greater at a height of 1.3 m.

### The EAA method

The EAA method [18] examines interactions between "focal" trees, made up of all the surviving trees during a census period in the FDP, and the "annular" trees that occupy successive concentric annuli of equal area around the focal trees. The use of these successive annuli, which each consist of similar amounts of data that can be analyzed with the same statistical power, permits unbiased statistical comparisons of the significance of interactions over a range of physical focal-annular tree distances.

EAA draws on many previous studies that have employed quadrat or point pattern analysis, coupled with a null modeling or Monte Carlo approach to generating control distributions in which specific components of the data have been randomized [11, 24–26]. The EAA method is an extension of neighborhood density functions such as the O-ring spatial statistic [26] and the Dx index [27]. EAA is similar to bivariate mark correlation analyses [26, 28]. The *r*-mark function is a non-parametric estimator of the response of the growth of small trees to the presence of a large tree at distance *r*. Point pattern methods are not based on regression analysis, like many of the methods that have been used to search for positive or negative density-dependent effects in forest data. These regression-based methods have recently been criticized as susceptible to over- or underestimation of the magnitudes of the effects that are being searched for [21]. EAA, in contrast, compares the distributions of the actual data with distributions in null models in which only the variable or variables of interest are repeatedly randomized and all other parameters of the FDPs are left untouched. Each iteration of the null model is analyzed in the same way as the real data, and the entire distribution of these null model results is used to test the difference between the real and randomized data. In each of these iterated replicates of the null-model data, any heteroskedasticity of the distributions of within-species focal tree growth rates and focal tree sizes is left unaltered and therefore cannot bias the results.

### A diagrammatic example of EAA analysis

Fig 1 shows, in diagrammatic form, the steps of a typical EAA analysis, in this case the relationship between focal tree size and clustering of annular trees in the Lambir FDP (Sarawak, Bornean Malaysia).

### Overview of the EAA tests

Table 2 lists the current EAA tests, the focal and annular tree properties that each test examines, and the expected results if focal-annular NDD interactions are present. Each of the tests is carried out as illustrated in Fig 1. Additional details of each test, including details of the null models used, are given below.

**Table 2:** Summary of the properties of, and expectations for, the EAA analyses that are used in this paper. The expected results for each test are for NDD focal-annular interactions; the PDD expectation is the opposite.

Focal tree properties	Annular tree properties	Null Model Comparison	Expected Results
**Focal-annular properties employed, and expectations shared by all tests:**			
Species, position, diameter, growth rate, recruitment and mortality for all trees in each census period	For each annulus: species, basal area, distance from focal tree in Ma, whether trees were recruited or died during census period	A single focal-tree attribute is repeatedly shuffled within species to serve as a control	Focal-annular differences from null model should decline in significance as either physical or phylogenetic focal-annular distance increases
**Properties examined and expect results for each null model comparison test, assuming NDD focal-annular interactions:**			
**Test 1) Relationship between focal survivor sizes and their annular survivor summed basal area**			
Focal survivor size	Summed basal area of annular tree survivors that fall within a given quantile of phylogenetic distance from focal tree	Shuffle focal tree survivor **sizes** within species	**Negative** relationship between focal tree size and annular survivor summed basal area
**Test 2) Relationship between focal survivor sizes and their annular recruit fraction**			
Focal survivor size	Fraction of trees in the annulus and phylogenetic distance quantile that recruit	Shuffle focal tree survivor **sizes** within species	**Negative** relationship between focal tree size and annular recruit fraction
**Test 3) Relationship between focal survivor sizes and their annular mortality fraction**			
Focal survivor size	Fraction of trees in the annulus and phylogenetic distance quantile that die	Shuffle focal tree survivor **sizes** within species	**Positive** relationship between focal tree size and fraction of annular trees that die
**Test 4) Relationship between a focal tree’s growth rate (normalized within species) and its annular tree basal area**			
Focal survivor growth rate	Summed annular survivor basal area in the phylogenetic distance quantile	Shuffle normalized focal tree survivor **growth rates** within species	**Negative** relationship between focal tree growth rate and annular tree summed basal area
**Test 5) Differences between focal trees that do and do not recruit and their annular recruit fractions**			
Focal recruits vs. other focal trees	Fraction of trees in the annulus and phylogenetic distance quantile that recruit	Shuffle properties of all focal trees within species	**Higher** fraction of annular recruits around focal recruits
**Test 6) Differences between focal trees that do and do not die and their annular mortality fractions**			
Focal trees that die (separated into small and large) vs. other focal trees	Fraction of trees in the annulus and phylogenetic distance quantile that die	Shuffle properties of all focal trees within species	**Higher** fraction of annular trees that die around focal trees that die

## Modifications of the original EAA method

### Overview of the modifications

The EAA method has been redesigned since its initial publication. In addition to the use of equal-area annuli surrounding focal trees, the method now divides sets of FDP data into equal quantiles, so that the statistical power of the analyses of all the subdivisions of the data in a given FDP are equivalent. Two- and three-dimensional GAM curves are now fit to the data. These curves reveal details and significance of effects traceable to phylogenetic distances between species. A uniform method of estimating phylogenetic distances between species is applied to all the FDPs.

Equations that quantify the analyses of focal-annular NDD-influenced effects are derived in [18]. Significances of the effects, compared to the mean of 1,000 iterations of the null models, are estimated using z-scores. The GAM analyses we employ here use spline-based smooth terms [29]. GAM fits of curves to the data use the formula y ~s(x, k = k-value), where s establishes the parameters of spline-based smooth terms and the k-value is the number of smooth terms employed. The optimal k-value is determined using gam.check [30]. Edge-effect corrections have been applied to all the FDPs [31]. The z-values obtained by all the analyses are adjusted for the discovery of false positives, using the Benjamini-Hochberg correction for independent statistics [32]. The data used to generate the GAM graphs are given in Supporting Information compressed data files S1 and S2 Datas.

### Division of the data into quantiles

Because we are comparing different FDPs that exhibit a wide range of species numbers, tree size distributions and densities, plot sizes, and distributions of phylogenetic distances between species, we have introduced standardized protocols for subdividing the data. Our goal is to ensure that each of the subdivisions of a set of data are of approximately equal size, so that the statistical power of the EAA tests remains the same across successive concentric annuli, phylogenetic distance intervals, and subdivisions of the focal and annular trees.

Annuli vary from 5 to 20 in number in the different FDPs, depending on overall tree density, but the total area around each focal tree encompassed by the annuli is 500 m^2^ in all FDPs. Focal tree diameters at the start of each census period for each species are divided into four equal quantiles. Totals of annular tree biomasses in each of the annuli (approximated by the sum of the areas at standardized height) are divided into five equal quantiles. Phylogenetic distances between species are also divided into quantiles, but this division poses special problems. First, the amount of data varies among FDPs. Therefore, in order to ensure that there is sufficient data for analysis, we have used different numbers of phylogenetic distance quantiles in different FDPs. We have been able to use as many as 20 quantiles in large, species-rich FDPs such as BCI and Pasoh, but have been limited to as few as 5 quantiles in smaller, less speciose FDPs such as Wind River and Wytham Woods. Second, because the distribution of pairwise phylogenetic distances between species is different in each FDP, and these distributions are far from uniform, subdivision of quantile differences often means that many species pairs that are separated by the same or similar phylogenetic distances will fall into different quantiles. Each analysis for each census period, therefore, is repeated ten times, each with 100 iterations of the null model. With each repetition, the order within each set of focal-annular pairs that share the same phylogenetic distance value is shuffled. This ensures that if large numbers of species pairs in an FDP share the same phylogenetic distance, subdivision into quantiles in the replicated analyses will have placed different random mixes of these pairs in adjacent quantiles.

### Measurement of effect of annular trees on focal tree growth rates

We define focal survivors as trees that are present at the beginning and end of a five-year census interval. For each data graph, data from all focal trees of all species are pooled. Normalized focal tree growth rates are calculated for each FDP as standard deviations from the mean of a given decile of diameters of the focal trees of a given species within a census period. This approach avoids the confounding effects of tree size differences, species differences, and secular trends over time on the growth rates of focal trees.

Summed annular tree basal areas are estimated as summed area at "breast height," the sum of the trees’ cross-sectional areas at a height of 1.30 m. The areas of multi-stemmed trees are summed. Basal areas and focal trees are each divided into five size quantiles. In the data presented in this paper, only the annular tree growth effects on the smallest size quintile of focal trees are examined, though as reported earlier there is a smaller but often significant negative effect of annular trees’ summed basal area on the growth of larger focal trees [18].

In these analyses, as in the analyses of recruitment, mortality, and annular tree clustering, each annulus is examined separately. Thus, an analysis of fifth-annulus annular tree effects on focal tree growth begins by examining all focal trees of the smallest diameter quintile. These focal trees either have, or do not have, annular trees in their fifth annulus that fall within a given quantile of Ma values to their last common ancestor (LCA) with the focal tree. Focal trees that have no such annular trees in their fifth annulus, regardless of whether or not they have such trees in their other annuli, form the comparison group. The focal trees that have such annular trees in their fifth annulus are divided into the five quintiles of summed annular tree basal areas and their growth rates are compared to those of the controls.

The null model used for comparison is generated by repeated randomization, within species, of the growth rates of the focal trees.

Annulus number and the number of phylogenetic distance quantiles used in each FDP analysis are adjusted to ensure that within each of the concentric annuli there will be a substantial number of such control annuli.

### Focal-annular phylogenetic distances

We estimated DNA-based divergences times between focal and annular species (last common ancestor (LCA) in mega-anna (Ma)) using the S.PhyloMaker program written by YJ (available at https://github.com/jinyizju/S.PhyloMaker). Table 3 presents the proportion of species in each FDP that are present in S.PhyloMaker’s Phytophylo DNA dataset. These proportions vary from 100% at Wind River to 14% at Sinharaja. The majority of phylogenetic distances between species must therefore be estimated at the genus rather than the species level in most of the FDPs. In this study the estimation was made by using Scenario 2 of S.PhyloMaker. For the species for which only genus-level information is known, this scenario picks uniformly-distributed distances from the interval from the present back to its genus’ LCA.

**Table 3:** Numbers and fraction of species in each of the FDPs in this study that are found in Phytophylo.

FDP	Species Found in Phytophylo	Species Not Found in Phytophylo	Fraction of Total Species found in Phytophyo
BCI	266	54	0.8313
Changbaishan	26	26	0.5
Fushan	45	66	0.4054
Gutianshan	117	42	0.7358
Heishiding	132	166	0.443
Korup	117	390	0.2308
Lambir	209	1127	0.1564
Luquillo	130	32	0.8025
Mudumalai	33	50	0.3976
Nonggang	82	135	0.3779
Palanan	49	267	0.1551
Pasoh	190	708	0.2116
Sinharaja	33	206	0.1381
Tiantong	105	51	0.6731
Windriver	17	0	1
Wytham	19	5	0.7917

There is unavoidably some noise in the phylogenetic distances, especially at FDPs such as Lambir and Pasoh where few species have been characterized genetically (Table 3). Further, and unavoidably, we are forced to add more noise because we divide the pairwise distances to the LCA into Ma interval quantiles that vary in number according to the amount of information in the FDP. This division has the advantage that it equalizes the amount of information in each quantile, but the disadvantage that it can add further noise to focal and annular species that are separated by pairwise distances that fall in a sparsely population region of the range of pairwise distances at the FDP. The noise problem can be overcome to some extent because we repeatedly sample the pairwise distances during the analysis. We have concluded that the advantage of having equal statistical power in each of the pairwise distance quantiles outweighs the disadvantage of introduced noise. This is because, when we have exhaustively analyzed FDPs more than once using this methodology, the results are essentially indistinguishable.

### Null models for clustering, recruitment and mortality

In order to isolate the influence of focal tree diameter on annular tree properties, the observed distribution of clustering, recruitment or mortality of annular trees around different diameter classes of focal trees is compared with 1,000 iterations in which focal tree diameters are randomized within species within census intervals in an FDP which is otherwise identical to the original FDP. Thus, these null models leave all other properties of the FDPs intact, including the positions and species identifications of all of the annular trees and the distributions of sizes of each species of focal tree. The only real-data components of clustering, recruitment and mortality that are measured are in the form of z-values of differences between the real data and the means of the Monte Carlo randomizations of focal tree diameters within species within census periods. This avoids the introduction of possible unknown variables, which is a problem with the regression analyses that are commonly used to search for density-dependent effects on recruitment and mortality [21]. Regression analyses search for differences in the properties of trees that surround trees that recruit or those that die, compared with those surrounding survivors, but the resulting regressions may have many sources traceable to the distributions of tree positions and tree properties that will vary across species.

### Null models and the influence of storage effects

There is a built-in “delay” in NDD-influenced factors that affect recruitment, mortality and clustering. This delay results from spatial and temporal storage effects [4, 6], allowing relatively dense clusters of trees of the same or phylogenetically related species to become established in regions where physical resources are initially plentiful and where species-specific pathogens, browsers, and seed-predators are initially few. As the trees in the clusters grow older and larger, their species-specific pathogens begin to accumulate, browsers and seed-predators become increasingly attracted to the area, and species-specific physical resources become limiting [33]. As a consequence, the trees in the clusters thin out over time, so that new clusters of saplings of the same species as those in the clusters can only be established elsewhere. By combining the original Janzen-Connell theory with spatial and temporal storage theory, it has been possible to explain the apparently contradictions between the Janzen-Connell model and the fact that many species of tree in forests are clustered rather than overdispersed [4, 6, 11]. Thus, null models that randomize only focal tree size and leave annular tree clustering or overdispersal intact are central to the EAA analyses, because these storage effects are the same in both the real and the randomized data.

### NDD- influenced focal-annular interactions

Many focal-annular interactions can be examined by the EAA method. We chose the interactions, listed below, that were shown in our previous study [18] to have an NDD component that remains significant across a wide range of focal-annular phylogenetic distances.

#### The effect of focal tree size on annular tree recruitment

NDD predicts that recruitment of annular trees at any phylogenetic distance from the focal tree should tend to be highest around small focal trees and diminish as the focal trees increase in size. Conditions favoring annular recruitment result from the accumulation of NDD effects of pathogens and parasites shared between focal and annular trees [8, 9], and from the depletion of shared physical and biologically-generated resources (niche-complementarity) [34, 35]. As focal trees grow, as shared pathogens accumulate, and as shared resources become scarcer, annular recruitment should diminish [3, 8, 9, 19]. Seedling data are not available for these data sets, and we therefore use the fraction of annular trees that have achieved a diameter of 1 cm during a census period as a proxy for recruitment rates.

#### The effect of focal tree size on annular tree mortality

The same NDD processes should result in low mortality among the annular trees that surround small focal trees and high mortality among annular trees that surround large focal trees [8, 9, 36].

#### The effect of focal tree size on annular tree clustering

The combination of NDD effects influencing recruitment and mortality should over time result in high summed basal area of annular trees around small focal trees and lower summed basal area of annular trees around large focal trees, again across a wide range of focal-annular phylogenetic separations.

#### The effect of annular tree basal area on focal tree growth

If a tree’s growth rate is slowed by the effects of competition for physical resources with nearby conspecific or phylogenetically related annular trees, or through the sharing of pathogens and predators with these annular trees, there may be a negative impact on its fitness [37]. Trees growing in regions that have few related trees nearby exhibit a fitness advantage over those growing in regions where there are many related trees nearby [24].

As noted above, and as in the original EAA analyses [18], focal tree growth rates are normalized within species, censuses, and focal tree size classes.

### Additional analyses

Other analyses based on comparisons of the actual data with randomized null models may be applied to these data. For example, the focal trees that recruit or die during a census period can be examined to determine the fraction of their annular trees that are also recruits or trees that die (tests 5 and 6 of Table 2). The null models for these tests determine the same ratios after the positions of the focal trees that recruit, survive and die are repeatedly shuffled within species and the properties of the annular trees are left untouched. These tests, however, only examine how interactions between focal trees that recruit or die and their annular trees that recruit or die, which are small fractions of all the focal-annular interactions, differ from those of the remainder of the focal-annular interactions.

### Interpretation of the graphical results

In interpreting the graphs of tests for these classes of focal-annular interactions, recall that in EAA analyses the z-values for the annular tree properties express differences from null-model data sets that are randomized only with respect to focal tree size. The z-values from the four quantiles of focal tree sizes are therefore symmetrically distributed around the mean of all the z-values.

Unlike the results for clustering, recruitment and mortality, the growth results are not symmetrically distributed around zero. Instead, each line shows the difference in z-scores between (a) the normalized growth rates of small focal trees that have, in a specified annulus, a specified summed basal area of annular trees within a specified quantile of phylogenetic distances from the focal tree, and (b) the normalized growth rates of small focal trees with no such annular trees in the specified annulus. In general, throughout the FDPs, the larger the summed basal area of the specified subset of annular trees, and the closer to the focal tree they are in either physical or phylogenetic distance, the greater the expected negative effect of the annular trees on normalized focal tree growth.

### A test for a possible relationship between tree size and patterns of tree mortality in this study

Mortality varies across life cycle, with NDD effects being most pronounced in the smallest trees [38]. NDD patterns of seedling mortality are primarily mediated by fungal pathogens [20, 39]. Mammals, foliar herbivores and foliar pathogens tend to contribute little to mortality at these early stages [40]. Phylogenetic distance is known to play an important role in the incidence of seedling mortality, which decreases as the phylogenetic distance between focal and surrounding trees increases [41–43].

The smallest trees for which mortality is measured in the present study have a dbh of 1 cm. We hypothesized that such small trees are more likely to die if they are near large focal trees, and less likely to die if they are near small focal trees. Larger trees that die might show a weaker association with focal tree size, because there might be a greater influence on the mortality of large trees of factors such as wind, fire and large herbivores that may have a small NDD component. To test this possibility, we divided annular trees of each species that died into two equal-sized groups, designated small and large.

### A control manipulation of the BCI data to check the sensitivity of the EAA analyses

Fig 2 below shows that focal-annular phylogenetic curves undergo the expected changes when FDP data are deliberately manipulated. EAA analyses are therefore highly sensitive to small differences in the data sets.

A pronounced reduction in significance in the BCI FDP data (marked with a circled numeral 1 at slightly over 100 Ma focal-annular phylogenetic distance in Fig 2 below) indicates that focal-annular species pairs separated by this phylogenetic distance are only interacting at low levels. This “valley” in significance values is seen clearly in focal-annular clustering, recruitment and the mortality of large annular trees. It is not apparent in mortality of small annular trees, or in focal tree growth.

To investigate the validity of this signal, we determined the set of phylogenetic distances between pairs of species at this FDP that lie between 108 and 117 Ma, and selected the interactions involving the three commonest species that are separated by these distances for our sensitivity test. Our reasoning was that, because these species are common in the plot, they are likely to contribute disproportionately to between-species interactions. The three, in descending order of abundance, are *Trichilia tuberculata* (Meliaceae) (5.0% of the stems), *Mouriri myrtilloides* (Melastomataceae) (3.4%), and *Tetragastris panamensis* (Burseraceae) (2.5%). The focal-annular phylogenetic distances to their LCA that fall in this window are *T*. *tuberculata* with *M*. *myrtilloides* (111 Ma) and *T*. *panamensis* with *M*. *myrtilloides* (111 Ma), while *T*. *tuberculata* with *T*. *panamensis* (72 Ma) falls outside the window.

We modified each of the focal-annular phylogenetic distances separating these three species to 50 Ma, and repeated the EAA analysis. The right-hand set of graphs in Fig 2 show the appearance of a new region of lowered significance (indicated with an asterisk) close to the 50 Ma mark for the clustering, recruitment and large-tree mortality graphs. The new lowered-significance signal does not appear precisely at 50 Ma, because these altered between-species distances now join a large group of other distances that contribute to the phylogenetic distance quantile that includes the 50 Ma distance. The original “valley” in significance values remains, though somewhat reduced in size, demonstrating that additional species pairs contribute to it. The growth graph shows no new signal, however, suggesting that the causes of the reduction in between-species interactions do not influence focal tree growth. Selective removals of species or focal-annular data from the data sets, such as those which were carried out at Wind River that are presented below, are therefore capable of providing consistent and detailed information about the extent of individual between-species interactions.

## Results

Each of the 16 forest diversity plots (FDPs) examined in this study exhibits smooth declines across increasing physical focal-annular distance (in m) in the significance of each of the four focal-annular interactions tested. There is one exception: recruitment at Mudumalai shows complexity across physical distance, possibly because of recent influence of elephants and/or fires (S1 Fig). Clustering at Mudumalai, which measures the results of longer-term processes, shares with the other FDPs the common pattern of a smooth decline with increasing physical distance.

These interactions also tend to show an overall decline in significance with increasing phylogenetic distances (in Ma) between the species, but there are many localized exceptions to this decline that result in unique patterns of “peaks” and “valleys” in the magnitude of significance along the phylogenetic distance axis for each FDP.

Table 4 summarizes the expected (from Table 2) and the observed results from the four EAA tests employed in this paper. In general, the results agree with NDD expectation, but there are many localized departures from a smooth decrease in significance with phylogenetic distance. In addition, there is a puzzling weak PDD signal seen for small annular trees that die and that warrants further investigation.

**Table 4:** Expected and observed results for the EAA tests, assuming NDD focal-annular interactions.

Expected (from Table 2)	Observed		
**Results expected and observed in all tests:**			
Focal-annular differences from null model should decline in significance as either physical or phylogenetic focal-annular distance increases	Smooth decline in significance with increasing physical distance. A general decline as phylogenetic distance increases, but many FDPs show significant peaks and valleys		
**Results expected and observed in each test, given NDD expectation:**			
**Test 1) Relationship between focal survivor sizes and their annular survivor summed basal area**			
**Negative** relationship between focal tree size and annular survivor summed basal area		Generally **negative,** as predicted, but there are some localized switches into PDD (positive) territory in some FDPs	
**Test 2) Relationship between focal survivor sizes and their annular recruit fraction**			
**Negative** relationship between focal tree size and annular recruit numbers			Generally **negative,** as predicted. Peaks and valleys along the phylogenetic axis often match annular survivor curves
**Test 3) Relationship between focal survivor sizes and their annular mortality fraction**			
**Positive** relationship between focal tree size and numbers of annular trees that die			**Positive,** as predicted for large annular trees that die, but weakly **negative** for small annular trees that die
**Test 4) Relationship between a focal tree’s growth rate (normalized within species) and its annular tree basal area**			
**Negative** relationship between focal tree growth rate and annular tree summed basal area		**Negative** relationship, as predicted. Significance falls with decreasing annular summed basal area, with many significant peaks and valleys along the phylogenetic axis	
**Test 5) Relationship between focal trees that do and that do not recruit. and their annular recruit fractions**			
**Higher** fraction of annular recruits around focal recruits		At BCI, a **higher** fraction, as predicted. Peaks and valleys along the phylogenetic axis do not match those for surviving focal trees	
**Test 6) Relationship between focal trees that do and do not die and their annular mortality fractions**			
**Higher** fraction of annular trees that die around focal trees that die		At BCI, a **higher** fraction, as predicted, for both small and large focal trees that die	

In Fig 3 we present the results, for each of the first four NDD-influenced focal-annular interactions that are listed in Table 2, at the BCI (Panama) FDP. The results are presented as two- and three-dimensional graphs generated from generalized additive model (GAM) analyses. The three-dimensional graphs in the figure present data from GAM analyses along both the physical (focal-annular distance in meters) and phylogenetic (Ma back to the last common ancestor (LCA)) axes, permitting a comparison of the effects of physical and phylogenetic distance in a single graph. Note that in these 3D graphs the physical distance decline remains smooth across all concentric annuli, while the irregularities in the phylogenetic distance curve are preserved across all concentric annuli. The smooth and gradual decline in significance with increasing physical focal-annular distance is clearly distinguishable from the complex fluctuations in significance that are seen across the range of phylogenetic distances. Figs S1 and S2 show that these patterns are seen in 3D analyses across FDPs, with the exception of recruitment at Mudumalai that was noted earlier.

Fig 4 shows phylogenetic distance results from a preliminary application of Tests 5 and 6 to the data from BCI.

The results are highly significant and in agreement with NDD expectation (Table 4, Tests 5 and 6). As with the other analyses, there is a smooth decline with increasing focal-annular physical distance (not shown), and a complex decline with increasing phylogenetic distance. The curves for mortality are in agreement with those shown for large annular tree mortality in Fig 3. However, the phylogenetic significance decline for recruitment appears to show a different pattern of hills and valleys from the equivalent analysis in Fig 3, suggesting that focal-annular interactions of focal trees that recruit or die with annular trees that recruit or die may be different from those of focal survivors. In addition, the phylogenetic peaks and valleys for recruitment are less complex than those found in the Fig 3 analysis, possibly because in Test 5 data are being examined from a smaller fraction of the focal trees. As these and other tests are explored further, they will provide additional windows of opportunity for investigation of detailed focal-annular interactions.

First-annulus two-dimensional graphs for Tests 1–4 in the remaining fifteen FDPs, which illustrate each FDP’s unique phylogenetic distance patterns, are shown in Figs 5, 6 and 7. NDD-influenced focal-annular interactions for clustering and recruitment are present in all of the FDPs, and NDD-influenced focal tree growth interactions with the summed basal areas of annular trees are also found in 15 of the FDPs.

Three-dimensional GAM graphs for clustering, recruitment and focal growth in all of the FDPs are shown in S1and S2 Figs. These three-dimensional graphs show that, with the exception of recruitment at Mudumalai, the significance of NDD-related effects declines smoothly rather than irregularly with increasing physical distance, while the patterns of peaks and valleys along the phylogenetic axis that are unique to each FDP are preserved across the range of physical focal-annular distances. These idiosyncratic phylogenetic distance patterns show that the species in each of the FDPs have in the past been shaped by distinct evolutionary trajectories that have led to this wide variety of patterns of species-species interactions.

Some of the exceptions to a smooth decline in significance with increasing focal-annular phylogenetic distance are observed at the same phylogenetic distances of an FDP’s recruitment, clustering, and/or focal tree growth analyses. In Figs 3 and 5–7, which present first-annulus 2D graphs for all sixteen FDPs, the most pronounced of these exceptions are marked with circled numbers 1 or 2 to mark significant local reductions or increases in significance respectively. Fig 6 shows a particularly striking example in the subtropical Luquillo FDP. At this FDP a valley and a peak, centered at 100 and 150 Ma back to the LCA respectively, are seen in clustering, recruitment and growth. If such exceptions are found at the same phylogenetic distance in more than one type of focal-annular interaction, they may have underlying causes in common (see the detailed analysis of the Wind River patterns below). Note that these resemblances are not the result of correlations in the numbers of recruits and survivors in the annuli, because these correlations are preserved in the null-model data to which the real data are compared (Materials and Methods).

### Mortality results show a more complex and difficult-to-interpret pattern

Thirteen of the sixteen FDPs show significant deviations from the null model in the pattern of small annular tree mortality. The pattern, however, is the opposite of that seen in previous studies of seedling mortality [20, 39]. More small annular trees than expected die around small focal trees, and fewer die than expected around large focal trees. This pattern, which is consistent with positive rather than negative density dependence, is largely confined to conspecific annular trees, except at the Nonggang, Tiantong and Wind River FDPs at which the PDD effect extends to some heterospecifics. This test is different from the BCI analysis of Fig 4 (d), which shows that more annular trees than expected die near the focal trees that die regardless of their size. The Fig 4 (d) analyses are consistent with NDD effects, in which mortality—especially mortality among closely-related trees—is expected to be spatially clustered.

In Figs 3, 5, 6 and 7 the mortality pattern exhibited by large annular trees that die is the reverse of that seen in the small annular trees, and like the Fig 4 (c) analysis is consistent with NDD effects. In fifteen of the FDPs, with the exception of the dry tropical forest plot at Mudumalai, there is a deficiency of mortality in large annular trees around small focal trees and an excess around large focal trees, as NDD would predict. We explore some possible reasons for the different outcomes of these different mortality tests in the Discussion.

### Known between-species interactions account for some of the variation in significance levels along the phylogenetic distance axis at the Wind River FDP

At the Wind River (Washington State, USA) FDP, which has only 26 tree species, EAA analysis reveals an unusually large exception to declines of NDD effects as focal-annular phylogenetic distance increases. At the largest focal-annular phylogenetic distances in this FDP, there are large increases in the significance of deviations of annular clustering and recruitment, and a large negative effect on focal tree growth rate. First-annulus data are used in the analysis presented in Fig 7, but as with the other FDPs these unique patterns of phylogenetic peaks and valleys at Wind River are preserved across more distant annuli.

A likely factor contributing to the unusual EAA pattern found in this FDP may be allelopathic inhibition. Large-diameter trees of the western hemlock *Tsuga heterophylla* have dense canopies and have been shown to have allelopathic needles [23]. These properties reduce the growth rate of trees of other species when *T*. *heterophylla* are nearby, and also reduce clustering and recruitment of trees of a range of species around large *T*. *heterophylla* [23, 43].

The effects of removing different combinations of species from the Wind River EAA analysis are shown in Fig 8. Trees of the commonest species, the vine maple *Acer circinatum*, and those of the second most common species, *Tsuga heterophylla*, are close to each other in numbers. Together, these two species make up two-thirds of the stems, and those of all the other species make up the remainder. We therefore divided the species into three categories: *A*. *circinatum*, *T*. *heterophylla*, and a third category made up of all the other species. We examined the effects of removing different combinations of focal or annular trees, or both, that fall into these three categories.

The row of graphs (1) at the top of Fig 8 show (A) annular clustering, (B) annular recruitment, and (C) focal tree growth GAM-generated curves for all the Wind River first-annulus data. All three of these focal-annular interactions show increases in significance among species separated by 150 Ma or more from their LCA, in the direction expected from NDD models. This is in striking contrast to the patterns of declining significance with increasing focal-annular phylogenetic distance that would be predicted if the strength of focal-annular interactions decreases with increasing phylogenetic distance between them. For focal growth, however, a group of species pairs that are separated by a little more than 100 Ma shows strong positive effects of annular trees on the growth of focal trees, consistent with PDD.

The next row of graphs (2) show results from only focal *T*. *heterophylla* and all annular species except for *A*. *circinatum*. The anomalous results at the most distant phylogenetic intervals are retained for clustering and recruitment, but disappear for focal tree growth (as does the equally anomalous PDD peak at more intermediate distances). This is the pattern to be expected if focal *T*. *heterophylla* are suppressing recruitment of distantly-related annular species (predominantly Angiosperms), but if the presence of these distantly-related annular species is not affecting the growth of the focal *T*. *heterophylla* in either a positive or a negative direction.

Graphs in row (3) show the results for only focal *A*. *circinatum* and all annular species except for *T*. *heterophylla*. These graphs present only the interactions between focal trees of the commonest species in the plot and annular species that are not known to have allelopathic effects. These interactions show a general decline of NDD-influenced effects with increasing phylogenetic distance across all phylogenetic distances. Thus, at Wind River, such a pattern—seen, though with many localized exceptions, at most of the FDPs—is revealed when only the interactions between focal trees of the commonest species *A*. *circinatum* and all annular species except for *T*. *heterophylla* are analyzed. When the entire data set is examined, however, this pattern is masked because of the strong allelopathic effects of *T*. *heterophylla*.

Row (4) conditions are the same as in Row (3), except that *T*. *heterophylla* has been added back to the annular trees. Clustering and recruitment patterns around focal *A circinatum* are little changed from Row (3), showing a lack of interactions between annular *T*. *heterophylla* and focal *A*. *circinatum*. There may be a small negative effect of *T*. *heterophylla* on the growth of focal *A*. *circinatum*, but it is at the margin of significance.

The graphs in Row (5) show results from the dataset consisting of all focal species except for *T*. *heterophylla* and all annular species except for *A*. *circinatum*. These results are the mirror image of Row (2). Clustering and recruitment of annular species are not influenced by allelopathy from focal trees other than *T*. *heterophylla*, and therefore do not show enhanced effects at extreme phylogenetic distances. But growth of distantly related focal species is slowed, as expected, by the allelopathic effects of annular *T*. *heterophylla*. And the striking peak in growth of intermediate-distance focal trees is again apparent, strongly indicating that annular *T*. *heterophylla* may be responsible for this effect as well.

Taken together, these results show that *T*. *heterophylla* influences at least some distantly related species negatively, and may influence trees at intermediate phylogenetic distances positively, but it does not influence the commonest species *A*. *circinatum*. Removal of other combinations of focal and annular species yields results that are consistent with this interpretation (not shown).

Previously-published measurements of the allelopathic effects of *T*. *heterophylla* had not detected *A*. *circinatum*’s immunity to *T*. *heterophylla*’s effects [23, 43].

The positive effects on focal growth seen in rows (1) and (4) of Fig 8 suggest that *T*. *heterophylla* has positive density-dependent effects on the growth of focal trees separated from it by intermediate phylogenetic distances. This interesting result warrants further investigation.

## Discussion

The FDPs examined here span a wide range of environments, from tropical to northern temperate. Across this diversity of ecosystems, the EAA results reinforce the growing body of evidence that NDD interactions between even distantly related species are common [44]. Here we provide a brief summary of conclusions that can be drawn and questions that can be raised from EAA analyses.

### Peaks and valleys in the significance of focal-annular species interactions along the phylogenetic axes for each FDP may result from unusual rates of evolutionary divergence and convergence between pairs or groups of species

For example, a valley in significance of annular tree effects on focal tree growth might result from the convergent evolution of distantly-related species on the ability to obtain resources from the environment [45]. A peak in significance in the NDD component of recruitment of distantly-related annular trees around focal trees might result from convergent evolution that has led to susceptibilities to similar pathogens [41] or browsers [46], while a valley in these significance levels between distantly-related focal and annular trees might result from divergent evolution in these susceptibilities. Evolution of allelopathy and of other offensive or defensive mechanisms in particular species, as in the Wind River FDP (Fig 8), must also play an important role in generating peaks and valleys.

We emphasize that the evolutionary changes leading to the peaks and valleys that we observe are not likely to have originated in the FDPs being examined, but must have had a much longer history of natural selection that took place in the various ecosystems that were inhabited by these species’ ancestors. Regardless of these anomalies’ precise origins, EAA can detect the between-species interactions that are most significant and that are therefore most likely with further study to yield useful information about the species’ evolutionary histories.

### Mortality interactions that have an NDD component may be confounded with effects that do not have an NDD component

Mortality of seedlings is known to have a strong NDD component [41, 42]. On the assumption that factors unrelated to NDD effects might play an important role in the mortality of large trees (such as large herbivores, strangler figs, windstorms, etc.), we divided the focal trees of each species that died in each FDP into two equally numerous groups (small and large) according to their diameters. When Test 3 of Table 2 was applied, the mortality pattern differences in these two groups were striking and unexpected.

For small annular trees that die, most of the FDPs show patterns consistent with *positive* density-dependence. There is low mortality around the largest focal trees and high mortality around the smallest focal trees. The FDPs Changbaishan and Palanan show no significant effects. In contrast, when the larger annular trees that die are examined, a positive density-dependent pattern is only seen at Changbaishan. Instead, a pattern that is consistent with NDD effects is seen at BCI, Fushan, Gutianshan, Heishiding, Korup, Lambir, Nonggang, Sinharaja, Tiantong, Wytham Wood and possibly Wind River. Examination of the causes of small-tree mortality may reveal the source or sources of the conspecific PDD effects.

### Storm-damaged FDPs show a variety of EAA patterns

The FDPs Palanan, Fushan and Luquillo are repeatedly damaged by cyclonic wind storms. These three FDPs have recently been compared with each other and with BCI, and were shown to have significant differences in mortality, growth and recruitment [47]. The present study found that all four FDPs also show differences in NDD patterns of mortality, growth and recruitment (Fig 6).

Palanan is a species-rich tropical forest in the Philippines that has been battered by three supertyphoons and numerous other storms during the 18 years that it has been censused. (It was hit with another supertyphoon in 2018, with effects yet to be measured.) This FDP shows weak focal-annular interactions that do not decline with increasing phylogenetic distance.

Although Luquillo is at the same latitude as Palanan and was damaged by two severe hurricanes during the period covered by this study, it shows a different set of focal-annular interactions. Luquillo, like Palanan, exhibits weak clustering and recruitment NDD effects that persist across most phylogenetic distances, but unlike at Palanan these effects diminish markedly at the largest distances.

Fushan, at a higher latitude, is a similarly storm-battered submontane FDP. It shows an interaction pattern more typical of the majority of FDPs, with strong interactions among conspecifics and an overall decline (with some dramatic exceptions) with increasing focal-annular phylogenetic distance.

Palanan and Luquillo have lower tree densities than Fushan, even though Fushan lies further north. In part this is the result of the more severe effects of the storms at Palanan and Luquillo, which often rip away the tops of canopy trees. These events open up the areas around large trees to higher levels of successful recruitment of all species [48, 49]. Enhanced recruitment may help explain why NDD-associated recruitment and clustering patterns are weakly significant and often change little over most phylogenetic distance in these FDPs. But at Luquillo, the reduction in the significance of clustering and recruitment at extreme phylogenetic distances is more consistent with the majority of FDPs.

### The three-dimensional GAM graphs show the persistence of peaks and valleys in species-species interactions across a range of physical distances

The unique shapes of the three-dimensional surfaces along their phylogenetic distance axis are retained across annuli at each FDP (Fig 3 and S1 and S2 Figs). They are retained even in distant annuli in which the means of the z-values that the surfaces represent are not themselves significantly different from zero (gray regions of the 3D surfaces). The surfaces can retain their phylogenetic-distance shapes, even in the gray regions, because the 95% confidence limits on the surfaces are small. As noted above, the rotatable three-dimensional GAM graphs from Fushan, Lambir and Pasoh that are presented in S3–S11 Figs allow the viewer to assess the relationships between the surfaces and their confidence intervals. In each case these intervals are small compared to the confidence intervals of the z-values themselves. Thus, the GAM analyses of the EAA data are able to detect the details of focal-annular interactions, even if the z-values themselves may be below the level of significance.

### Species with different properties do not on average contribute disproportionately to the results

In the first EAA paper [18] it was shown for the BCI FDP that between-species NDD interactions are of approximately equal strength across a variety of groupings of species into subsets that have different phenotypic and ecological properties. In that analysis it was decided not to divide species into phenotypic classes that would have led to unequal-sized subdivisions, because this would have made the tests for NDD-influenced patterns less directly comparable to each other. To preserve the statistical power of the tests, and to make them comparable to each other, the BCI species were subdivided using two different criteria. The first criterion sorted the species according to their abundance, and the second sorted them according to the CV of their diameters across all the stems of the species. After ranking of the species according to each of these criteria, the pooled trees of the ranked species were then divided into thirds, each third consisting of equal numbers of individual trees. EAA analyses of each of these six subdivisions showed that all six exhibited the same EAA NDD patterns as the BCI FDP as a whole, though as expected the subdivision results were less significant than they were in the total data. Thus, in this FDP, EAA analysis of species with a wide range of properties yields similar results.

Detailed examinations of NDD interactions between conspecifics of individual species of different abundances at BCI, however, have suggested substantial differences in the strength of NDD-influenced patterns, to the point that the least-influenced species may be in danger of local extinction [50]. EAA can be used to investigate the effects of the removal of information about individual species that have similar abundances in an FDP, in order to test this observation further.

### EAA analyses can be used to investigate ecological-evolutionary processes in detail

It is possible to use EAA to examine interactions between focal-annular species pairs that are based, not only on the phylogenetic distance between them, but also on other quantifiable characteristics: differences in the species’ physical and biochemical phenotypes, in their defensive and allelopathic mechanisms, in their shared interactions with different classes of pathogens, herbivores and parasites, and in their associations with the plots’ topographies and soil types. The only requirement for such an analysis is the ability to arrange focal-annular species pairs on a scale of numerical values for the character, from least divergent to most divergent.

The EAA approach can then be modified to "sieve out" focal-annular species combinations that show the greatest discordance between phylogenetic distance and such scalable phenotypic and environmental characteristics. A large focal-annular phylogenetic distance, coupled with a small focal-annular distance in a simultaneously measured phenotypic or niche-related character, would suggest evolutionary convergence in the character being measured, while the reverse situation would suggest an unusually high rate of evolutionary divergence. This approach will permit the isolation of the characteristics that are most likely to be associated with cases of unusual focal-annular divergence or convergence in FDPs and in similar complex ecosystems. The nature of these interactions can be explored further by experimental manipulations in the field or in greenhouse experiments.

Most of the thousands of species in these plots are rare. Their aggregate contributions to NDD effects may be substantial, but most of these contributions are unlikely to be detectable at the species level. EAA provides us with a tool for finding the species that are likely to repay further study, while still applying the rigorous EAA standard of examining the effects of only one variable at a time.

The EAA approach may also be sensitive enough to detect small changes over time in the focal-annular dynamics of multiply-censused FDPs such as BCI and Pasoh that may be correlated with climatic change.

### EAA and Darwin’s hypothesis

EAA analyses can be used to test an important ecological-evolutionary prediction that follows from Darwin’s observations and from his speculations that were quoted at the beginning of this paper. When species have lived in close physical proximity to each other within an ecological community for some time, some of these species’ evolutionary changes should have been driven by the direct and indirect interactions among them. Even if particular pairs of tree species are not physically close in the ecosystem, they will share symbionts, pollinators, pathogens, parasites, predators, and herbivores that often have high levels of dispersal. These shared biological agents must also have undergone evolutionary change as a result of their interactions with their hosts. It is likely that the variety of evolutionary trajectories in the NDD-influenced focal-annular effects that are seen in the FDPs of this study stems in part from such biotic interactions. EAA can detect the pairs of tree species in an FDP that are likely candidates for detailed studies of these interactions, which will in turn help towards eventual clarification of their true causes and their evolutionary histories. At the same time, such extended studies will reveal the proportion of these interactions that are responses to physical factors in the species’ environment, and provide firm evidence for or against Darwin’s hypothesis that biological factors play a large role in ecosystem evolution.

### EAA and ecosystem preservation

In order to preserve threatened ecosystems, we must understand the mechanisms that maintain their diversity. EAA can be used to flag between-species interactions that are unusually strong or weak and are therefore likely to yield significant results when subjected to experimental manipulation. Each such case that is understood in depth will increase our understanding of the kinds of interactions that must be preserved in order to maintain the overall structure of both intact and endangered ecosystems. General ecological models that ignore these interactions are of little help in understanding which aspects of ecosystems are important in their long-term preservation.

## Supporting information

S1 Fig Three-dimensional graphs showing the differences between the physical (m) and phylogenetic (Ma) axes for z-values that measure the significance of the NDD-influenced component or annular tree clustering and recruitment for the sixteen FDPs. The graphs show the patterns seen around the largest quantile of focal tree sizes. Legends and surface colors as in Figs 1 and 3. Note that levels of significance decrease smoothly with increasing focal-annular physical distance and irregularly with increasing focal-annular phylogenetic distance at each FDP. In addition, the shapes of the phylogenetic distance curves for clustering and recruitment often resemble each other, for reasons discussed in the Results section of the main paper.
(TIF)

S2 Fig Three-dimensional graphs showing the effect of the largest quintile of annular trees on the growth of focal trees for all 16 FDPs. Legend as in Figs 1 and 3.
(TIFF)

S3 Fig bci_focal_growth_largest_annular_biomass_type_factor.html. An interactive rotatable graph of a three-dimensional GAM analysis of focal growth data from the BCI FDP. The graph allows the viewer to examine the surfaces formed by the data from all vantages, in order to visualize the differences between the physical and the phylogenetic distance axes. The areas of the surface on the graph that lie in regions of significant z-values are shown in color. The areas that lie in regions of non-significant z-values are in gray. Because of the size of the graph, it is possible to show clearly the magnitude of the 95% confidence intervals of the surface itself, which tend to be small. The surface with its confidence interval also shows that irregular features along the surface’s phylogenetic distance axis retain their shapes and their significance even in parts of the surface that lie within regions where the individual z-values themselves are non-significant.
(HTML)

S4 Fig bci_annular_clust_largest_focal_trees_type_factor.html. An interactive rotatable graph of a three-dimensional GAM analysis of clustering data from the BCI FDP. Legend as in S3 Fig.
(HTML)

S5 Fig bci_annular_born_largest_focal_trees_type_factor.html. An interactive rotatable graph of a three-dimensional GAM analysis of recruitment data from the BCI FDP. Legend as in S3 Fig.
(HTML)

S6 Fig fushan_focal_growth_largest_annular_biomass_type_factor.html. An interactive rotatable graph of a three-dimensional GAM analysis of focal growth data from the Fushan FDP. Legend as in S3 Fig.
(HTML)

S7 Fig fushan_annular_clust_largest_focal_trees_type_factor.html. An interactive rotatable graph of a three-dimensional GAM analysis of clustering data from the Fushan FDP. Legend as in S3 Fig.
(HTML)

S8 Fig fushan_annular_born_largest_focal_trees_type_factor.html. An interactive rotatable graph of a three-dimensional GAM analysis of recruitment data from the Fushan FDP. Legend as in S3 Fig.
(HTML)

S9 Fig luquillo_focal_growth_largest_annular_biomass_type_factor.html. An interactive rotatable graph of a three-dimensional GAM analysis of focal growth data from the Luquillo FDP. Legend as in S3 Fig.
(HTML)

S10 Fig luquillo_annular_clust_largest_focal_trees_type_factor.html. An interactive rotatable graph of a three-dimensional GAM analysis of clustering data from the Luquillo FDP. Legend as in S3 Fig.
(HTML)

S11 Fig Luquillo_annular_born_largest_focal_trees_type_factor.html. An interactive rotatable graph of a three-dimensional GAM analysis of recruitment data from the Luquillo FDP. Legend as in S3 Fig.
(HTML)

S1 Data Data for FDP’s Mudumalai, Wytham Woods, Heishiding, Nonggang and Palanan. (ZIP)

S2 Data Data for remainder of FDP’s in the study. These zipped files contain all the data used to plot the figure graphs, including information needed to generate the errors on the graphs, in .csv format.
(ZIP)
