# Supplementary figures and images for: Interactions between all pairs of neighboring trees in 16 forests worldwide reveal details of unique ecological processes in each forest, and provide windows into their evolutionary histories
Source: PLoS Comput Biol. 2021 Apr 29;17(4):e1008853. doi: 10.1371/journal.pcbi.1008853 (PMC8084225; doi:10.1371/journal.pcbi.1008853)

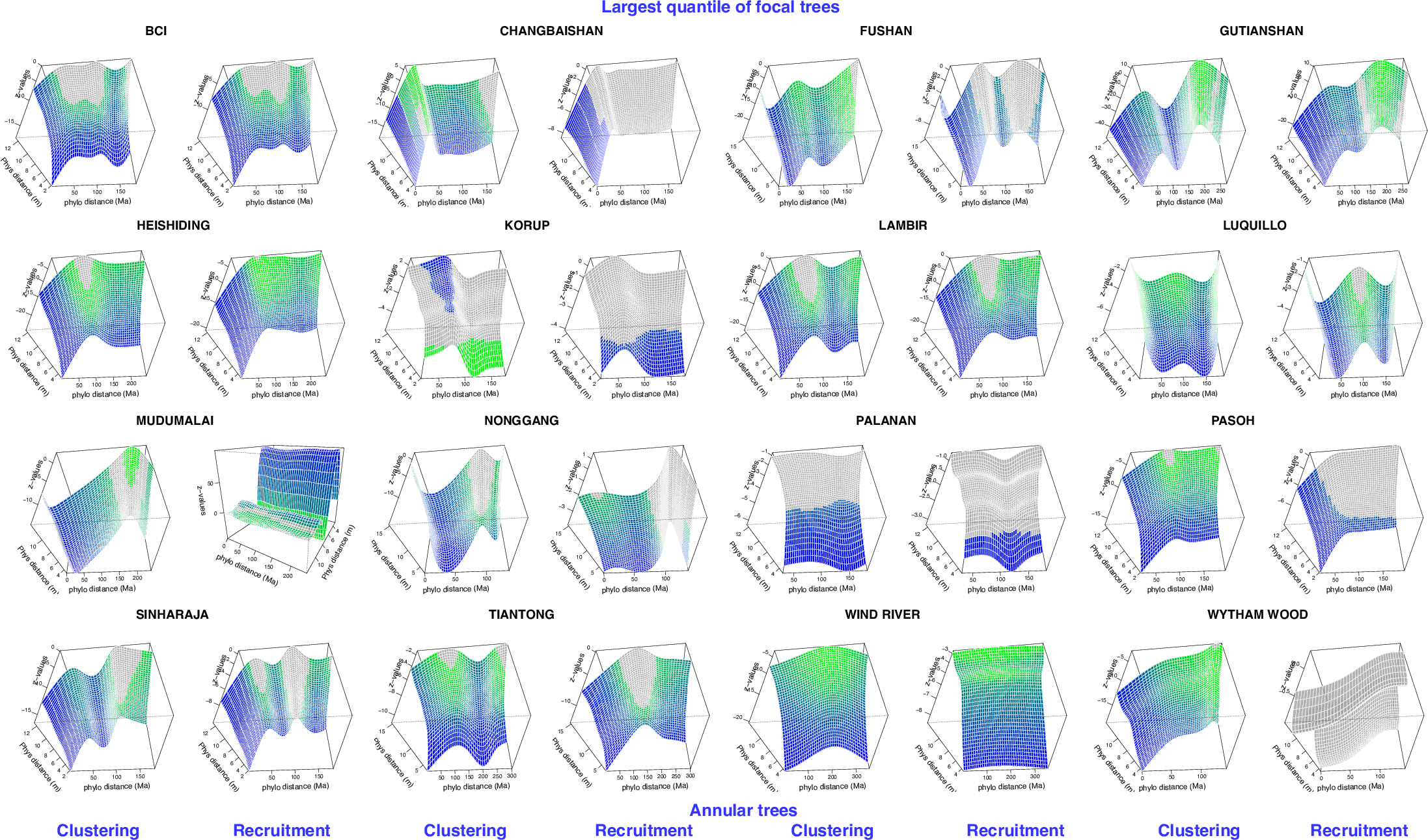

Supplement: S1 Fig — The graphs show the patterns seen around the largest quantile of focal tree sizes. Legends and surface colors as in Figs 1 and 3. Note that levels of significance decrease smoothly with increasing focal-annular physical distance and irregularly with increasing focal-annular phylogenetic distance at each FDP. In addition, the shapes of the phylogenetic distance curves for clustering and recruitment often resemble each other, for reasons discussed in the Results section of the main paper. (TIF) [file pcbi.1008853.s001.tif]

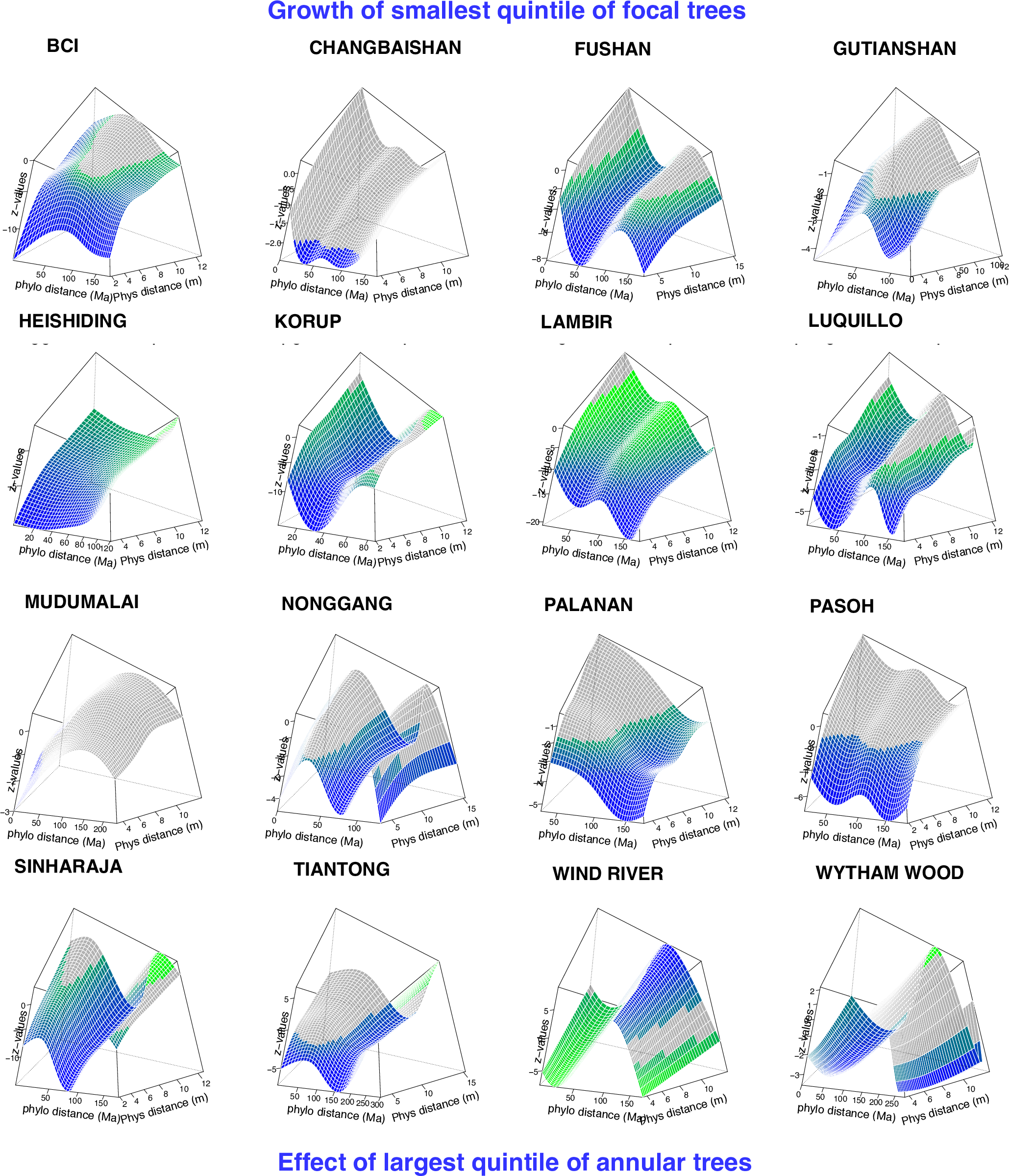

Supplement: S2 Fig — Legend as in Figs 1 and 3. (TIFF) [file pcbi.1008853.s002.tiff]
