# Supplementary material for: Interactions between all pairs of neighboring trees in 16 forests worldwide reveal details of unique ecological processes in each forest, and provide windows into their evolutionary histories
Source: PLoS Comput Biol. 2021 Apr 29;17(4):e1008853. doi: 10.1371/journal.pcbi.1008853 (PMC8084225; doi:10.1371/journal.pcbi.1008853)
Supplement: S3 Fig — An interactive rotatable graph of a three-dimensional GAM analysis of focal growth data from the BCI FDP. The graph allows the viewer to examine the surfaces formed by the data from all vantages, in order to visualize the differences between the physical and the phylogenetic distance axes. The areas of the surface on the graph that lie in regions of significant z-values are shown in color. The areas that lie in regions of non-significant z-values are in gray. Because of the size of the graph, it is possible to show clearly the magnitude of the 95% confidence intervals of the surface itself, which tend to be small. The surface with its confidence interval also shows that irregular features along the surface’s phylogenetic distance axis retain their shapes and their significance even in parts of the surface that lie within regions where the individual z-values themselves are non-significant. (HTML) [file pcbi.1008853.s003.html]

 
RGL model


You must enable Javascript to view this page properly.

  
Drag mouse to rotate model. Use mouse wheel or middle button
to zoom it.

---

  
Object written from rgl 0.100.19 by writeWebGL.
